# Modulation of Lipoteichoic Acids and Exopolysaccharides Prevents *Streptococcus mutans* Biofilm Accumulation

**DOI:** 10.3390/molecules25092232

**Published:** 2020-05-09

**Authors:** Midian C. Castillo Pedraza, Erick Dante de Oliveira Fratucelli, Sabrina Marcela Ribeiro, Elkin Jahir Florez Salamanca, Jaqueline da Silva Colin, Marlise I. Klein

**Affiliations:** Department of Dental Materials and Prosthodontics, School of Dentistry, Sao Paulo State University (UNESP), Rua Humaita, 1680, Araraquara 14801-903, Sao Paulo, Brazil; midianclar@gmail.com (M.C.C.P.); erickdante121@gmail.com (E.D.d.O.F.); sabrina.ribeiro@unesp.br (S.M.R.); ej.florezsalamanca@gmail.com (E.J.F.S.); jakescolin@live.com (J.d.S.C.)

**Keywords:** biofilm, *Streptococcus mutans*, compound 1771, myricetin, exopolysaccharides, lipoteichoic acids

## Abstract

Dental caries is a diet–biofilm-dependent disease. *Streptococcus mutans* contributes to cariogenic biofilms by producing an extracellular matrix rich in exopolysaccharides and acids. The study aimed to determine the effect of topical treatments with compound 1771 (modulates lipoteichoic acid (LTA) metabolism) and myricetin (affects the synthesis of exopolysaccharides) on *S. mutans* biofilms. In vitro *S. mutans* UA159 biofilms were grown on saliva-coated hydroxyapatite discs, alternating 0.1% sucrose and 0.5% sucrose plus 1% starch. Twice-daily topical treatments were performed with both agents alone and combined with and without fluoride: compound 1771 (2.6 µg/mL), myricetin (500 µg/mL), 1771 + myricetin, fluoride (250 ppm), 1771 + fluoride, myricetin + fluoride, 1771 + myricetin + fluoride, and vehicle. Biofilms were evaluated via microbiological, biochemical, imaging, and gene expression methods. Compound 1771 alone yielded less viable counts, biomass, exopolysaccharides, and extracellular LTA. Moreover, the combination 1771 + myricetin + fluoride decreased three logs of bacterium counts, 60% biomass, >74% exopolysaccharides, and 20% LTA. The effect of treatments on extracellular DNA was not pronounced. The combination strategy affected the size of microcolonies and exopolysaccharides distribution and inhibited the expression of genes linked to insoluble exopolysaccharides synthesis. Therefore, compound 1771 prevented the accumulation of *S. mutans* biofilm; however, the effect was more pronounced when it was associated with fluoride and myricetin.

## 1. Introduction

Dental caries is a public health problem worldwide affecting humans without distinction of age and is the main factor related to the loss of dentition [1]. Several factors interact for disease occurrence [2], and its development is associated with the transition from a healthy biofilm to a pathogenic one driven by a high-sugar diet [3,4]. Thus, strategies are needed to control cariogenic biofilm development.

Among several species that comprise the oral microbiota, *Streptococcus mutans* is considered an important etiological factor for dental caries in association with a sugar-rich diet [4,5]. The uniqueness of this species is its ability to orchestrate the cariogenic biofilm build-up by producing a three-dimensional (3D) extracellular matrix rich in exopolysaccharides (EPS) that hampers diffusion and creates microenvironments where metabolites such acids accumulate within the biofilm and at the interface biofilm/teeth surface [6], leading to the demineralization of teeth enamel [7]. This matrix also contains extracellular DNA (eDNA) and lipoteichoic acid (LTA) [8,9]. This bacterium also produces acids and withstands acidic niches [10].

Dietary sucrose is a substrate and starch hydrolysates can serve as acceptors for EPS synthesis by *S. mutans* exoenzymes. The glycosyltransferases (GtfB, GtfC, and GtfD) present on the pellicle and microbial surfaces produce glucans and enabling the local accumulation of microbes on the teeth [11]. These exoenzymes produce distinct glucans while GtfB produces water-insoluble glucans (mainly α 1,3 linkages), GtfD produces water-soluble glucans (mainly α 1,6 linkages), and GtfC produces both water-soluble and -insoluble glucans. The insoluble glucans are the main components of the cariogenic biofilm extracellular matrix. Furthermore, fructans produced by a fructosyltransferase are a minor part of EPS in the matrix [11]. Over time, these EPS build a matrix that embeds the microbial cells forming a cohesive and diffusion-limiting milieu, creating acidic niches where cariogenic species thrive and cause acid-dissolution of teeth [6,7]. Moreover, EPS-rich extracellular matrix protects the microorganisms from antimicrobial therapies and provides viscoelasticity to biofilms, hindering its mechanical removal [11].

eDNA cooperates with EPS in the early phases of biofilm development while LTA participates in the maturation of matrix and biofilm [9]. The deletion of the *gtfB* gene affects the expression dynamics of eDNA-linked genes (*lytST*, *lrgAB*, *ccpA*) [12]. It markedly decreases the expression of *dltB* (from operon *dltABCD* part of the LTA metabolism pathway) and EPS-associated genes (*gtfBCD, gbpB, dexA*) [12]. Most of those genes have gene products involved with cell wall/membrane composition/structure, indicating that these cell components turnover may be affected, thereby influencing matrix and biofilm development [12]. The inactivation of *lytST*, *dltAD*, and *gtfB* impaired *S. mutans* cariogenicity in a rodent model, and its virulence in a systemic infection model (*Galleria mellonella* larvae) [13]. Hence, strategies to modulate these genes and their products could affect *S. mutans* pathogenicity.

Classical caries prevention strategies include exposure to fluoride (aid in the remineralization process), restriction of dietary sugars (reduce substrate for microbial EPS and acid production), and mechanical removal of dental plaque (brushing and flossing). In addition, chlorhexidine, a broad-spectrum bactericidal agent, suppresses mutans streptococci levels in saliva but is less effective against biofilms and has undesirable side effects [14]. Nevertheless, fluoride is the mainstay for caries prevention as it has led to a significant reduction in decay; however, it offers incomplete protection against caries and has minimal effect on microbial aciduricity and acidogenicity, even though it can affect the glycolytic activity of streptococci [15]. Extensive efforts for fluoridated drinking water have been in place, but it does not reach the entire population, especially in Brazil [16]. Moreover, promoting fluoride toothpaste would retard caries in populations but would not eliminate it, as caries occur in populations with good fluoride exposure [17]. Additionally, an increase in daily dosage may lead to fluorosis of teeth and bones [18,19].

The use of fluoride combined with bioactive agents can improve the action of fluoride and become an interesting strategy for cariogenic anti-biofilm therapies. In this context, myricetin is a flavonoid that inhibits *gtfBC* expression and EPS synthesis [20,21,22]. This flavonoid also affects *S. mutans* acidogenicity by hindering the F-ATPase activity and glycolytic pH-drop [20]. Furthermore, the compound 1771 is a small molecule that inhibits the synthesis of LTA in Gram-positive bacteria *Staphylococcus aureus* [23] and *Enterococcus faecium* [24]. Thus, here it was investigated whether the combination of myricetin, compound 1771, and fluoride influences *S. mutans* biofilm development by interfering with the bacterium gene expression and metabolism that would hamper the extracellular matrix build-up and consequent bulky cariogenic biofilm.

## 2. Material and Methods

### 2.1. Experimental Design 

An in vitro study with *Streptococcus mutans* was conducted to investigate the antimicrobial and antibiofilm activity of compound 1771 and myricetin. The antimicrobial activity was evaluated using planktonic cultures in microdilution assay to determine the minimum inhibitory concentration (MIC) and minimum bactericidal concentration (MBC). Next, *S. mutans* single-species biofilm based on two batch culture approaches were used to investigate the anti-biofilm activity. First, the MIC and 2xMIC concentrations were used to evaluate the anti-biofilm effect of each agent using a polystyrene microplate biofilm model by determining *S. mutans* viable population (colony forming units or CFU/biofilm) and biomass (crystal violet method). After, 2xMIC of both agents alone and combined with and without fluoride were used for twice-daily topical treatments of biofilms formed on saliva-coated hydroxyapatite discs as substrate. The study was approved by the Institutional Ethical Committee (CAAE: 31717914.3.0000.5416).

In the second biofilm model, the eight experimental groups that corresponded to the distinct topical treatments were: compound 1771 (2.6 µg/mL), myricetin (500 µg/mL), compound 1771 + myricetin, fluoride (250 ppm), compound 1771 + fluoride, myricetin + fluoride, compound 1771 + myricetin + fluoride, and vehicle (3.2% ethanol plus 0.02% dimethyl sulfoxide—DMSO). After 67 h, biofilms were evaluated to determine biofilm biomass, *S. mutans* viable counts, and extracellular matrix components (water-soluble and -insoluble EPS, eDNA, and LTA). Furthermore, confocal laser scanning microscopy was used to visualize and quantify the structural components (EPS and bacteria) of the biofilms. Furthermore, after 46 h of biofilm development, gene expression of the selected genes was performed. Moreover, during biofilm growth, the culture medium was changed twice daily, and the pH values of the spent medium were measured. 

Three independent experiments were carried out in triplicate for the antimicrobial and anti-biofilm on polystyrene surface (n = 9), while in duplicate for the anti-biofilm on saliva-coated hydroxyapatite surface (n = 6). The data were statistically analyzed according to the factorial design of this study, considering each microplate well or hydroxyapatite disc as a statistical block. The discs were randomly assigned for each experimental group. Biochemical analyses of the extracellular matrix were performed to verify whether specific agents or combinations of agents influenced biofilm development and consequent pathogenic potential. Our hypothesis was that interfering with EPS synthesis and cooperation with eDNA and LTA by using selected bioactive agents would hinder the development of *S. mutans* cariogenic biofilm. In addition, the cytotoxicity of each treatment and vehicle was investigated.

### 2.2. Test Agents

Compound 1771 ([(5-phenyl-1,3,4-oxadiazol-2-yl)carbamoyl]methyl 2-{naphtha [2,1-b] furan-1-yl}acetate) and myricetin were acquired via MolPort ordering service; compound 1771 was supplied by UkrOrg Synthesis Ltd. (Cat. # PB25353228) while myricetin was supplied by AK Scientific Inc (Cat. # J10595). Sodium fluoride was purchased from Sigma-Aldrich Co. (St Louis, MO, USA; Cat. # 71519). Stock solutions of each agent were prepared. Compound 1771 was dissolved in the combination of 84.5% ethanol and 15% DMSO at a final concentration of 2 mg/mL (4.68 mM). Myricetin was dissolved in 84.5% ethanol, with 1xPBS (*Phosphate Buffered Saline*, pH 7.2) at a final concentration of 15.912 mg/mL (50 mM).

For the antimicrobial activity assays, the concentrations used were 166.7−0.3255 μg/mL for compound 1771 and 500−3.9 μg/mL for myricetin. For the antibiofilm assays on the polystyrene surface, the concentrations used were MIC and 2xMIC. The concentrations selected for twice-daily topical treatments of biofilms formed on saliva-coated hydroxyapatite discs as substrate were 2xMIC of both agents, alone combined with and without fluoride: myricetin or Myr (500 µg/mL), compound 1771 or 1771 (2.6 µg/mL), compound 1771 + myricetin or Myr + 1771, myricetin + fluoride (250 ppm) or Myr + F, compound 1771 + fluoride or 1771 + F, compound 1771 + myricetin + fluoride or Myr + 1771 + F, fluoride or F (250 ppm), and vehicle or V (3.2% ethanol plus 0.02% DMSO). Thus, for the topical treatments, the test agents, including fluoride, were dissolved in 3.2% ethanol containing 0.02% DMSO just before carrying out the assays. The concentration of fluoride was selected based on the commercially available fluoride-based mouthrinse [22,25].

### 2.3. Bacterial Strain and Growth Conditions

*S. mutans* UA159, serotype c (ATCC 700610) strain stocks were stored at −80 °C in tryptic soy broth containing 20% glycerol and were plated on blood agar plates (37 °C, 5% CO_2_/95% air atmosphere, 48 h). Starter cultures were prepared by inoculating 5 to 10 colonies in tryptone-yeast extract broth (TY; 2.5% tryptone, 1.5% yeast extract, pH 7.0) containing 1% glucose tryptone broth and 1% glucose yeast extract, followed by incubation for 16 h (37 °C, 5% CO_2_/95% air atmosphere). The starter cultures were diluted 1:20 in fresh TY + 1% glucose and incubated until mid-exponential growth phase for the antimicrobial and antibiofilm assays.

### 2.4. Antimicrobial Activity

The antimicrobial activity was evaluated by determining the minimum inhibitory concentration (MIC) and minimum bactericidal concentration (MBC) using broth microdilution following the Clinical and Laboratory Standards Institute [26], with some modifications. The *S. mutans* cultures at the mid-exponential growth phase were diluted in TY + 1% glucose until reaching 2 × 10^6^ CFU/mL. Aliquots of 100 µL of these cultures were transferred to 96-well microplates. Next, treatments or vehicle were added, so the final bacterial load was 1 × 10^6^ CFU/mL, and the microplates were incubated (37 °C, 5% CO_2_/95% air atmosphere, 24 h). In addition to treatments and the vehicle, a microbial growth control without treatment was included; controls per treatment without microbial inoculation were also performed. Each treatment was performed in triplicates at three distinct experiments. After 24 h of incubation, visual inspection of the wells (turbidity: microbial growth, clear: no growth) and OD_562_ nm readings (ELISA plate reader, Biochrom Ez, Cambourne, UK) were performed to determine MIC. The MIC should be the agent concentration in which all wells were clear. Furthermore, an aliquot of each well was used for a 10-fold serial dilution (10^0^ to 10^−5^) in saline solution (0.89% NaCl; Synth) to determine the viability of the microbial cells by plating blood agar plates (37 °C, 5% CO_2_/95% air atmosphere, 48 h). After incubation, the colonies were counted to obtain CFU/mL and determine the MBC. The MBC should be the agent concentration in which no bacterial growth was observed on agar plates.

### 2.5. Antibiofilm Assay Using the Polystyrene Microplate Model

The initial anti-biofilm assay was performed using the polystyrene microplate model to investigate whether compound 1771 presented antibiofilm activity. Even though it is known that myricetin has this activity [20,21,22], it was tested here to ensure that the one from the acquired here also had this property. Therefore, *S. mutans* cultures at the mid-exponential growth phase were diluted in TY + 1% sucrose until reaching 2 × 10^6^ CFU/mL. Aliquots of 100 µL of these cultures were transferred to 96-well microplates. Next, treatments (MIC and 2xMIC) or vehicle were added, so the final bacterial load was 1 × 10^6^ CFU/mL, and the microplates were incubated (37 °C, 5% CO_2_/95% air atmosphere, 24 h). In addition to treatments and the vehicle, a microbial growth control without treatment was included; controls per treatment and medium without microbial inoculation were also performed. After 24 h of incubation, the microplates were placed on an orbital shaker at 75 rpm for 5 min (Quimis, São Paulo, Brazil). Next, the culture medium with unbound microbial cells was removed, and remaining biofilms were washed three times, with 0.89% NaCl solution to remove non-adhered cells.

The biomass of treated biofilms was assessed via the crystal violet method. Briefly, the washed biofilms were stained with an aqueous solution of 0.1% crystal violet and incubated (25 °C, 35 min). Next, the wells were washed using Milli-Q water and air-dried for 60–90 min. Then, the crystal violet was eluted with 99% ethanol and incubation for 5 min on the orbital shaker (37 °C, 200 rpm). The eluted volumes were transferred to another microplate, and the OD_570_ nm was measured on an ELISA plate reader (Biochrom Ez, Cambourne, UK). The readings per treatment and controls were converted into the percentage of biofilm biomass inhibition by each treatment and compared to vehicle control.

In addition, to verify the viable counts of the microbial population of treated biofilms, the washed biofilms were removed from each well using pipet tips and 0.89% NaCl solution and transferred to microtubes. Then, an aliquot of each biofilm suspension was used for a 10-fold serial dilution (10^0^ to 10^−5^) followed by plating on blood agar plates (37 °C, 5% CO_2_/95% air atmosphere, 48 h). After incubation, the colonies were counted to obtain CFU/mL and calculate the log of microbial growth inhibition by each agent, compared to vehicle control.

### 2.6. Biofilm Formation on Saliva-Coated Hydroxyapatite Discs and Topical Treatments

Biofilms of *S. mutans* UA159 were formed on saliva-coated hydroxyapatite (sHA) discs (surface area of 2.93 ± 0.2 cm^2^, Clarkson Chromatography Products Inc., South Williamsport, PA, USA) in batch cultures for 67 h, as detailed elsewhere [9]. Saliva and pellicle preparation were performed as described before [27]. Saliva was donated by two volunteers who had not used antimicrobial treatments in the last three months. Each volunteer rinsed their mouth with 5 mL of Milli-Q water, then masticate a piece of parafilm, collecting 5 mL saliva into a collection tube, which was then discarded. Next, the volunteers continued masticating the parafilm and collected saliva into an ice-chilled tube. The saliva samples from all volunteers were pooled and diluted 1:1 with adsorption buffer (AB buffer: 50 mM KCl, 1 mM KPO_4_, 1 mM CaCl_2_, 1 mM MgCl_2_, 0.1 mM PMSF, in dd-H_2_O, pH 6.5). Saliva was centrifuged (3220× *g*, 20 min, 4 °C; Centrifuge 5810R, Eppendorf), and the clarified portion was filtered sterilized (0.22 µm low protein binding polyethersulfone membrane filter). Saliva was used fresh for pellicle formation and medium preparation at the start of the experiment, and any remaining saliva was aliquotted and stored at −80 °C until use for culture media preparation.

Saliva-coated HA discs were placed in a vertical position in a 24 well microtiter dish using a custom-made disc holder [9]. Next, these discs with salivary pellicle were dip-washed into wells containing AB buffer and topically treated during 1.5 min with the seven tested treatments or vehicle control (eight experimental groups): (1) Myr; (2) 1771; (3) Myr + 1771; (4) Myr + F; (5) 1771 + F; (6) Myr + 1771 + F; (7) F; (8) V. The procedure for topical treatment consisted of dripping 250 µL of each treatment on the corresponding discs (two discs per treatment and experiment). The treated discs were dip-washed into wells containing AB buffer and transferred to wells containing *S. mutans* culture for biofilm formation (time 0 h). Here, the *S. mutans* cultures at the late-exponential growth phase were diluted in TY + 0.1% sucrose and 25% saliva until reaching 2 × 10^6^ CFU/mL. The biofilms were incubated for 6 h (37 °C, 5% CO_2_/95% air atmosphere), when the discs with initial biofilm were rinsed with 0.89% NaCl, treated with each corresponding treatment or control (as above), rinsed with 0.89% NaCl (to remove excess treatment), and transferred back to the corresponding culture media until biofilms were 19 h-old, when culture medium was changed.

**Figure 1:**
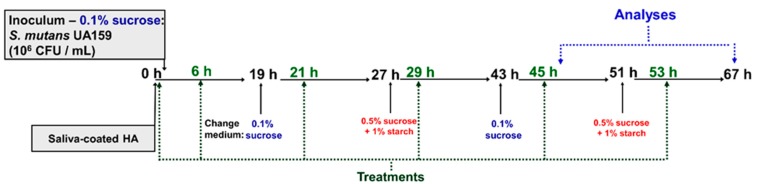
Experimental design for twice-daily topical treatments using a saliva-coated hydroxyapatite biofilm model. The treatments were performed at 0 (pellicle, before starting incubation with *S. mutans*), 6, 21, 29, 45, and 53 h of biofilm development. The biofilms were analyzed at 46 and 67 h. The pH of the spent medium was evaluated at 19, 27, 43, 46, 51, and 67 h.

The culture medium was changed daily at 8 a.m. (TY + 0.1% sucrose and 25% saliva) and 4 p.m. (0.5% sucrose + 1% starch and 25% saliva). After each media change, the pH of the spent media was measured. The biofilms were topically treated two hours after each culture change (Figure 1). The biofilms were grown until reaching two developmental phases: 46 h for gene expression assessment and 67 h for microbial population, biomass, biochemical characteristics of biofilm extracellular matrix, and confocal analyses.

### 2.7. Biofilm Analyses

#### 2.7.1. Determination of Microbial Population, Biomass and Biochemical Characteristics of Biofilm Extracellular Matrix

At 67 h of development, biofilms were processed for analyses following previously described protocols [27]. Briefly, biofilms were dip-washed into wells containing sterile 0.89% NaCl solution (saline solution). Each biofilm (disc) was transferred to a glass tube containing 1 mL of saline solution. Next, 1 mL of saline solution was used to wash the walls of each tube. The glass tubes with biofilms/discs were placed in a beaker and subjected to water-bath sonication for 10 min. A sterile metal spatula was used to scrape off any remaining biofilm from each disc surface, and the 2 mL of each biofilm suspension was transferred to a new 15 mL tube. Next, each glass tube was washed with 3 mL of saline solution, which was transferred to the tube containing the initial 2 mL, yielding 5 mL total biofilm suspension per biofilm/disc. Each biofilm suspension (5 mL) was sonicated using a probe at 7 w for 30 s (Sonicator model Q125, QSonica, Newtown, CT, USA).

An aliquot of each suspension (0.1 mL) was used for a 10-fold serial dilution to determine the number of CFU by plating on blood agar plates (37 °C, 5% CO_2_/95% air atmosphere, 48 h). The remaining volume (4.8 mL) was centrifuged (3220× *g*, 20 min, 4 °C). The supernatant (with soluble extracellular matrix components) was transferred to a new tube, and the pellet (precipitate with the microbial cells and insoluble matrix components) was washed twice with 2.6 mL sterile Milli-Q water (3220× *g*, 20 min, 4 °C). The supernatants generated during the two washes were combined with the first supernatant obtained, totaling 10 mL, which was used to isolate and quantify water-soluble EPS [28], eDNA [29], and LTA [9]. The pellet was suspended in 2.55 mL of Milli-Q water, of which 0.5 mL was used for quantification of insoluble dry-weight (biomass) and 1 mL for the isolation and quantitation of water-insoluble EPS (or alkali-soluble polysaccharides) [28].

#### 2.7.2. Laser Scanning Confocal Fluorescence Microscopy Imaging and Computational Analyses of Treated Biofilms

Biofilms were formed and treated as described above. However, 1 μM Alexa Fluor™ 647-labeled dextran conjugate (absorbance/fluorescence emission maxima of 647/668 nm; Molecular Probes, Carlsbad, CA, USA) was added to the culture medium at the beginning of, and during, development of the biofilms [30]. This strategy enables the incorporation of labeled dextrans into EPS during its synthesis process and matrix build-up. When the biofilms reached 67 h of development, the discs were dip-washed into wells containing with 0.89% NaCl and transferred to wells containing 0.89% NaCl solution and SYTO™ 9 (485/498 nm; Molecular Probes) which is a green fluorescent nucleic acid marker for visualization of bacteria [30]. The imaging of the three-dimensional structure of these biofilms was performed using a Zeiss LSM 780 microscope (Zeiss, Jena, Germany), fitted with a 20× objective lens. Each biofilm was scanned at three randomly selected positions, and a series of confocal images were generated by optical sectioning at each of these positions. The images were analyzed using Amira 6.0.1 software (Mercury Computer Systems Inc., Chelmsford, MS, USA) for 3D reconstruction of EPS and bacteria [6]. Furthermore, each image was analyzed using COMSTAT version 2 software [31] for quantification of total bacteria content and EPS matrix (bio-volume), and percent of coverage per area from the interface substrate/biofilm (hydroxyapatite disc) to the top (outer layer) of each biofilm.

#### 2.7.3. Gene Expression Analysis of Treated *S. mutans* Biofilms

Five treatments for biofilms were chosen to evaluate *S. mutans* gene expression based on the data from biochemical analyses and 3D structure of biofilms: Myr, 1771, Myr + 1771 + F, F, and V (control). The *S. mutans* assessed genes were associated with insoluble EPS (*gtfB* and *gtfC*), eDNA (*IrgA*), LTA (*dltB*, *dltD*, and *SMU.775*), and tolerance to acid (*atpD*) and oxidative stresses (*nox1*) [6,12]. Biofilms were grown and topically treated, as described earlier. At 46 h of growth that corresponds to 1 h after treatments, biofilms were removed and processed for total RNA isolation, followed by cDNA synthesis and quantitative PCR (qPCR) following the MIQE guidelines [32].

The biofilms were dip-washed into wells containing sterile 0.89% NaCl solution (saline solution). Each biofilm (disc) was transferred to a glass tube containing 1 mL of RNAlater™ Stabilization Solution (Ambion, Austin, TX, USA). Next, 1 mL of RNAlater™ was used to wash the walls of each tube. The glass tubes with biofilms/discs were placed in a beaker and subjected to water-bath sonication for 10 min for biofilm detachment from discs. A sterile RNAse-free metal spatula was used to scrape off any remaining biofilm from each disc surface. Each biofilm suspension was transferred to a new centrifuge tube, and two mL of RNAlater™ were used to rinse the glass and the discs; this volume was recovered and stored in the corresponding centrifuge tubes. These biofilm suspensions were stored at −80 °C until RNA isolation.

RNA was extracted as described elsewhere [33]. Briefly, biofilm suspensions in RNAlater™ were diluted 1:1 with 1xPBS (pH 7.2) and centrifuged (3220× *g*, 20 min, 4 °C). The supernatant was discarded, and the resulting pellet was resuspended in 5 mL of 1xPBS, followed by sonication with a probe for 30 s at 7 w (Sonicator model Q125); this procedure was repeated twice. After, the pellet was resuspended in 750 μL NAES (50 mM sodium acetate buffer, 10 mM EDTA and 1% SDS; pH 5.0; Ambion) and 750 μL acid phenol (Ambion). This mixture was transferred to tubes containing glass beads, followed by mechanical disruption using a Bead-beater for 40 s (Biospec Products) and cooling samples by placing them on ice for 1 min. This homogenization procedure was repeated twice, and the samples were centrifuged (15,480× *g*, 5 min, 4 °C; Centrifuge 5430R, Eppendorf). Then, RNA was extracted using the phenol-chloroform acid separation method and purified with DNAse in column (RNeasy Micro Kit, Qiagen, Austin, TX, USA) and solution (Turbo DNase; Ambion, Austin, TX, USA). DNAse was removed using the RNeasy MinElute clean-up kit (Qiagen). After purification, the total RNA amount (OD 260 nm) and purity (OD 260/280 ratio) were verified (DS-11 + Nano-spectrophotometer). The integrity of the purified RNA was determined by 1% agarose gel electrophoresis. RNA was diluted to a concentration of 100 μg/μL and stored at −80 °C until cDNA synthesis.

cDNA synthesis was performed in duplicate per sample using 0.5 μg of total RNA and the iScript kit (Bio-Rad Laboratories, Inc., Hercules, CA, USA). Negative controls were made without using reverse transcriptase to determine whether there was DNA contamination. Reactions were incubated using CFX96 Touch ™ Real-Time PCR Detection System (Bio-Rad), with the cycle: 25 °C/5 min, 42 °C/30 min, 85 °C/5 min, 4 °C ∞. The cDNA samples were stored at −20 °C until used for the quantification of gene expression (qPCR).

qPCR analyses were performed using specific primers from the literature [6,12]. cDNA was diluted 1:5 for specific genes and 1:1000 for the 16S rRNA gene (used for normalization of specific gene expression); cDNA negative controls were not diluted. cDNA and negative controls were amplified by a CFX96 System (Bio-Rad) using specific primers and iQ SYBR Green supermix (Bio-Rad). A standard curve was plotted for each primer set as detailed elsewhere [34]. The reactions were run using the following cycle in a CFX96 System (Bio-Rad) equipment: step 1 (1×) 95 °C/3 min; Step 2 (35×) 95 °C/15 s, 58 °C/30 s, 68 °C/15 s (data collection); Step 3 (1×) 95 °C/1 min; Step 4 (1×) 55 °C/1 min; Step 5 (80×) 55 °C/1 min, for analysis of the melting curve. The standard curves were used to transform the Quantification Cycle (Cq) values to relative numbers of cDNA molecules. Relative expression was calculated by normalizing each gene of interest to the 16S rRNA reference gene. Next, these values were compared to those from biofilms treated with vehicle-control to determine the fold-change in gene expression.

### 2.8. Cytotoxicity of Treatments

Oral keratinocytes NOK-si lineage [35] cells were grown in Dulbecco’s Modified Eagle’s Medium (DMEM, GIBCO, Grand Island, NY, USA) with 2 mM glutamine; containing 10% fetal bovine serum (FBS, GIBCO, Grand Island, NY, USA), penicillin G (10.000 μg/mL), streptomycin (10.000 μg/mL) and amphotericin (25 μg/mL) (Invitrogen). The culture was incubated (37 °C, 5% CO_2_/95% air atmosphere). The cells were grown to confluency (90%), washed with 1× phosphate buffer (140 mM NaCl, 3.0 mM KCl, 4.30 mM Na_2_HPO_4_, 1.40 mM KH_2_ PO_4_, pH 7.0), removed with trypsin (0.05%)/EDTA solution (0.53 mM) (Invitrogen), and then centrifuged (400× *g*, 5 min). The cells were resuspended in the same culture medium and replated. The medium was changed every two or three days. For the experiments, cells between the 3rd and 8th passages were used. Cells were counted in Neubauer’s chamber and plated in 96-well microplate wells (2 × 10^4^ cells well). The plates were incubated for 24 h (37 °C, 5% CO_2_/95% air atmosphere).

Next, the cytotoxicity resulting from the presence of treatments (agents and vehicle control), death control (0.11% Triton X-100), and untreated control (cell viability control) on monolayer cells was determined by the colorimetric assay of viability cell MTT [3-(4,5-dimethylthiazol-2-yl) 2,5-diphenyltetrazolium bromide] (Sigma). This assay was performed using cell culture in monolayer, 1 h after contact with treatments and controls included in the culture medium. After the incubation period, the cells were washed with 500 μL of 1xPBS (pH 7.4) and incubated (37 °C, 5% CO_2_/95% air atmosphere, 4 h) with 250 μL of MTT solution (5 mg/mL). Then the forming crystals were solubilized in 250 μL of 2-propanol added to each well. Spectrophotometric measurements were performed at a wavelength of 562 nm. Two experimental occasions were performed with 4 replicates per occasion (n = 8). The data obtained were converted into a percentage of viable cells and compared to the control without treatment (control of cell viability) [36].

### 2.9. Statistical Analyses

The data were analyzed to evaluate whether the tested treatments affected *S. mutans* biofilm using Prism 7 software (GraphPad Software, Inc., San Diego, CA, USA, 2018). The analyses were performed using descriptive and inferential statistics according to the distribution (Shapiro-Wilk test of normality; α = 0.05). The antimicrobial activity data of myricetin and compound 1771 showed a normal distribution and were evaluated with one-way ANOVA, Dunnett’s post-test (α = 0.05) (Supplementary Materials). The anti-biofilm activity data of myricetin and compound 1771 did not present normal distribution, so it was evaluated with the Kruskal-Wallis test, with Dunn’s post-test (α = 0.05). The data obtained for topically treated biofilms presented a normal distribution; thus, data for the viable counts, biomass, matrix components (water-soluble and -insoluble EPS, eDNA, and LTA), biovolume quantification of bacteria and EPS were evaluated by one-way ANOVA, followed by test Tukey’s multiple comparisons (α = 0.05). The gene expression data were normalized by the 16S rRNA gene data, followed by an analysis of the fold change relative to the vehicle. Data for gene *gtfB*, *gtfC*, *IrgA*, and *SMU.775* did not present normal distribution and were examined using the Kruskal–Wallis test, followed by Dunn’s post-test (α = 0.05). Meanwhile, data for genes *atpD*, *nox1*, *dltB,* and *dltD* presented a parametric distribution and were evaluated via one-way ANOVA, followed by test Tukey’s multiple comparisons (α = 0.05). The data from cytotoxic assays presented normal distribution and were evaluated using one-way ANOVA, followed by test Tukey’s multiple comparisons (α = 0.05).

## 3. Results

### 3.1. Antimicrobial Activity

**Figure 2:**
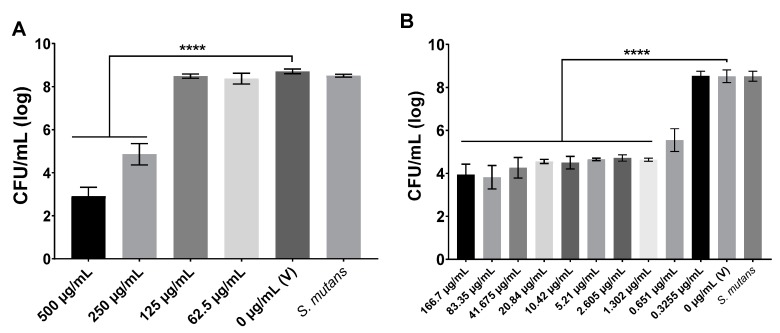
Antimicrobial activity of myricetin (**A**) and compound 1771 (**B**). The *S. mutans* viable population data are shown as average, and error bars correspond to the standard deviation (n = 12). V: Vehicle; for myricetin, the vehicle was 7% ethanol in 1xPBS (pH 7.2); while for compound 1771, it was 7% ethanol and 1.25% dimethyl sulfoxide (DMSO). *S. mutans*: growth control culture. **** denotes a statistically significant difference between the vehicle and the concentrations tested (*p* < 0.0001; one-way ANOVA test, followed by Dunnett’s multiple comparisons test).

The MIC value was 1.302 µg/mL for compound 1771, while for myricetin, it was 250 µg/mL. Figure 2 depicts the *S. mutans* viable population recovered at distinct concentrations of agents. As none of the tested concentration for both agents leads to the absence of bacterial growth on agar plates, the MBC was not determined. However, an effective antimicrobial agent concentration could the one that reduced the CFU/mL by at least three logs compared to the vehicle control.

### 3.2. Antibiofilm Activity Using the Polystyrene Microplate Model

**Figure 3:**
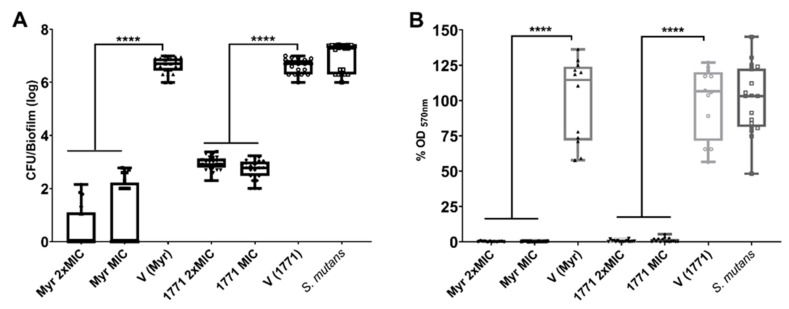
Antibiofilm activity of myricetin and compound 1771 tested at concentrations of minimum inhibitory concentration (MIC) and 2xMIC using the polystyrene microplate model. The *S. mutans* viable population (**A**) and biomass (**B**) data are shown as the median and interquartile range (n = 12). The two concentrations of the two agents were able to inhibit both viable population and biomass. The vehicle for each agent is shown separately because the experiments were not performed at the same time. *S. mutans*: growth control group. **** denotes the statistical difference between treatments and V (*p* < 0.0001; Kruskal–Wallis test, followed by Dunn’s post-test). Myr: myricetin. 1771: compound 1771. V: vehicle used for each compound.

The anti-biofilm effect of MIC and 2xMIC for compound 1771 and myricetin are shown in Figure 3. Both agents reduced the counts of *S. mutans* viable population by >4 logs and biomass by >99% (*p* ˂ 0.0001; Kruskal–Wallis test with Dunn post-test). Thus, both assays demonstrated that both agents could inhibit *S. mutans* biofilm. The difference in the magnitude of the effect in the distinct assays is because platting evaluated the viable population, and the biomass encompasses the microbial cells and the extracellular matrix.

### 3.3. Effect of Topical Treatments on Biofilms Grown on Saliva-Coated Hydroxyapatite Discs

#### 3.3.1. pH of the Spent Culture Media

**Figure 4:**
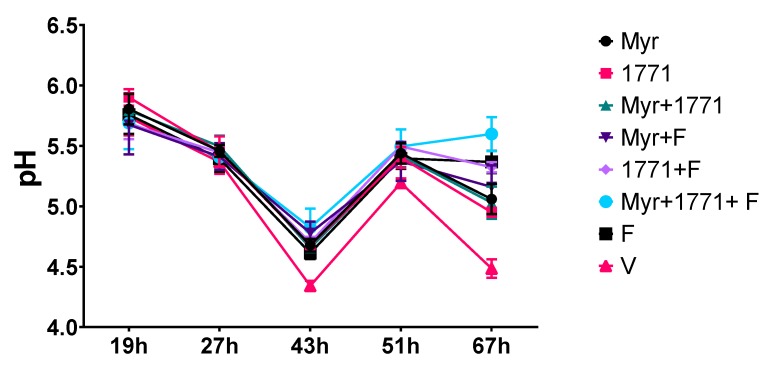
pH of spent culture media from topically treated biofilms at distinct developmental phases. The spent biofilm culture media were evaluated at 19, 27, 43, 51, and 67 h. At 19 h, the culture medium of biofilms treated with compound 1771 showed a higher pH value when compared to Myr + F (*p* = 0.0009), 1771 + F (*p* = 0.0017), Myr + 1771 + F (*p* = 0.0027), and V (*p* = 0.0466) (one-way ANOVA, followed by Tukey’s test). At 27 h, there was no significant difference between treatments. The culture medium from vehicle-treated biofilms showed lower pH at 43, 51, and 67 h compared to all treatments (*p* ˂ 0.0001; one-way ANOVA, followed by Tukey’s test). The medium from Myr + 1771 + F had the highest pH value when compared to Myr + 1771 and F at 43 h, and at presented the highest pH value at 67 h (*p* < 0.05, one-way ANOVA, followed by Tukey’s test). The data represented are the means, and the error bars correspond to the standard deviation.

The pH of spent media was measured after the culture medium changes (19, 27, 43, and 51 h) and when the biofilms were removed for processing and analyses (67 h) (Figure 4). At 19 h the biofilms treated with 1771 had the highest pH value (it was less acidic) when compared to the treatments Myr + F, 1771 + F, Myr + 1771 + F, and V (*p* < 0.05; one-way ANOVA, followed by Tukey’s test). At 27 h, there were no differences between treatments and vehicle. However, at 43, 51 and 67 h, vehicle group presented the lowest pH value versus all treatments (*p* ˂ 0.0001; one-way ANOVA, followed by Tukey’s test), while the combination strategy Myr + 1771 + F, yielded the highest pH value compared to all other treatments (*p* ≤ 0.0356; one-way ANOVA, followed by Tukey’s test). Additionally, at 67 h F treatment yielded the highest pH value when compared to Myr, 1771, Myr + 1771, and Myr + F (*p* ˂ 0.0001; one-way ANOVA, followed by Tukey’s test). Furthermore, the agents combined with fluoride lead to higher pH values in the spent media (vs. vehicle or agents alone or combined). In addition, the pH values were similar for all treatments at 46 h, when biofilms were processed for gene expression analyses (these data are not depicted in the graph because they are from five experimental groups). Of note, the ‘rise’ in pH values of the spent medium from 43 to 51 h is because, during that period, the biofilms were incubated in culture media with 0.1% sucrose.

#### 3.3.2. Viable Counts of *S. mutans* and Biomass

**Figure 5:**
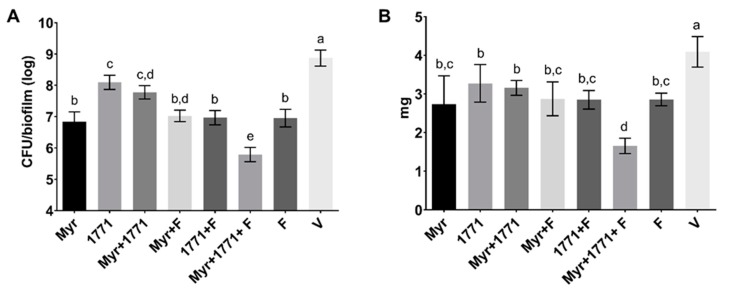
*S. mutans* viable counts (**A**) and insoluble biomass (dry-weight) (**B**) of topically treated biofilms. At 67 h, the vehicle yielded the highest values of CFU/biofilm and biomass, while the lowest values were found for the combination of Myr + 1771 + F. Bars with the same letters indicate no statistical difference between the different treatments in each graph (*p* > 0.05; one-way ANOVA, followed by Tukey’s test). The data represented are the means, and the error bars correspond to the standard deviation.

The quantification of viable *S. mutans* numbers is shown in Figure 5A. All treatment with agents alone or combined with or without fluoride reduced the bacterial population in comparison to the vehicle (*p* ˂ 0.0001; one-way ANOVA, followed by Tukey’s test). The compound 1771 alone reduced 1 log compared to the vehicle. However, this effect was most pronounced in the biofilms treated with the combination strategy Myr + 1771 + F, which had three logs difference from the vehicle-treated biofilm (*p* ˂ 0.0001; one-way ANOVA, followed by Tukey’s test). Furthermore, a similar trend was observed for the biomasses of the insoluble portion of *S. mutans* biofilms (Figure 5B); all treatments were able to decrease the values versus the vehicle-treated (*p* ˂ 0.0001; one-way ANOVA, followed by Tukey’s test). Still, the highest reduction in biomass was observed for biofilms treated with the combination of Myr + 1771 + F when compared to all other treatments and the vehicle (*p* ˂ 0.0001; one-way ANOVA, followed by Tukey’s test), showing a ≈60% reduction versus the vehicle.

#### 3.3.3. Extracellular Matrix Components

**Figure 6:**
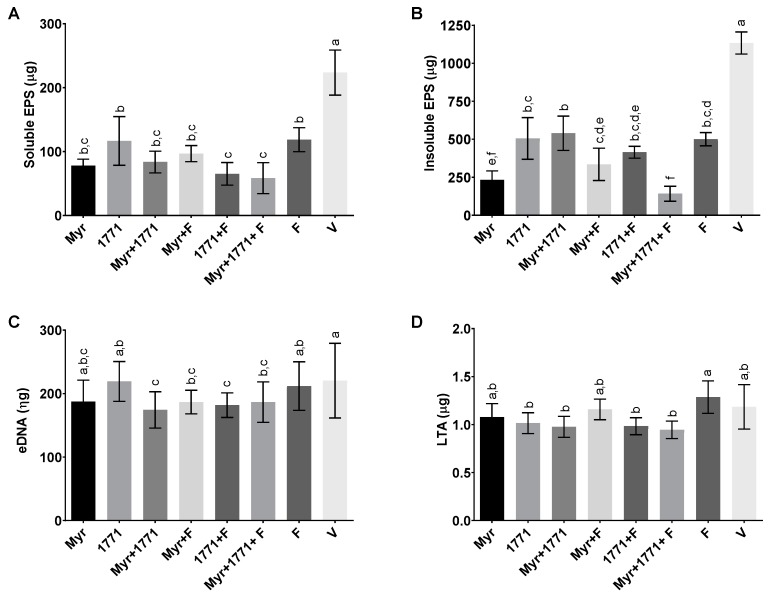
Modulation of the extracellular matrix components in the topically treated biofilms. The graphs exhibit the content of water-soluble exopolysaccharides (EPS) (**A**), water-insoluble EPS (**B**), eDNA (**C**), and lipoteichoic acids (LTA) (**D**) in the extracellular matrix. Bars with the same letters indicate no statistical difference between the different treatments in each graph (*p* > 0.05; one-way ANOVA, followed by Tukey’s test). The data represented are the means, and the error bars correspond to the standard deviation.

The quantification of four components of the extracellular matrix is depicted in Figure 6. Regarding EPS, all treatments yielded biofilms with lower quantities of both water-soluble and -insoluble EPS compared to vehicle-treated biofilms (Figure 6A,B; *p* < 0.0001; one-way ANOVA, followed by Tukey’s test). However, the combination of Myr + 1771 + F was more effective in hindering the accumulation of water-insoluble EPS (a marker of cariogenic biofilms; ≈87.87% ± 4.23%) and water-soluble EPS (vs. all other treatments; ≈73.88% ± 10.83%), although the effect of Myr alone was also pronounced for both types of EPS. In contrast, the effect of treatments on eDNA and LTA amounts was not noticeable (Figure 6C,D). Nevertheless, the four treatments containing compound 1771 yielded about 15% to 20% less LTA in the matrix than the other treatments, especially fluoride (*p* ≤ 0.0303; one-way ANOVA, followed by Tukey’s test).

#### 3.3.4. Structure, Biovolume and Percent Coverage Distribution of Bacteria and EPS

**Figure 7:**
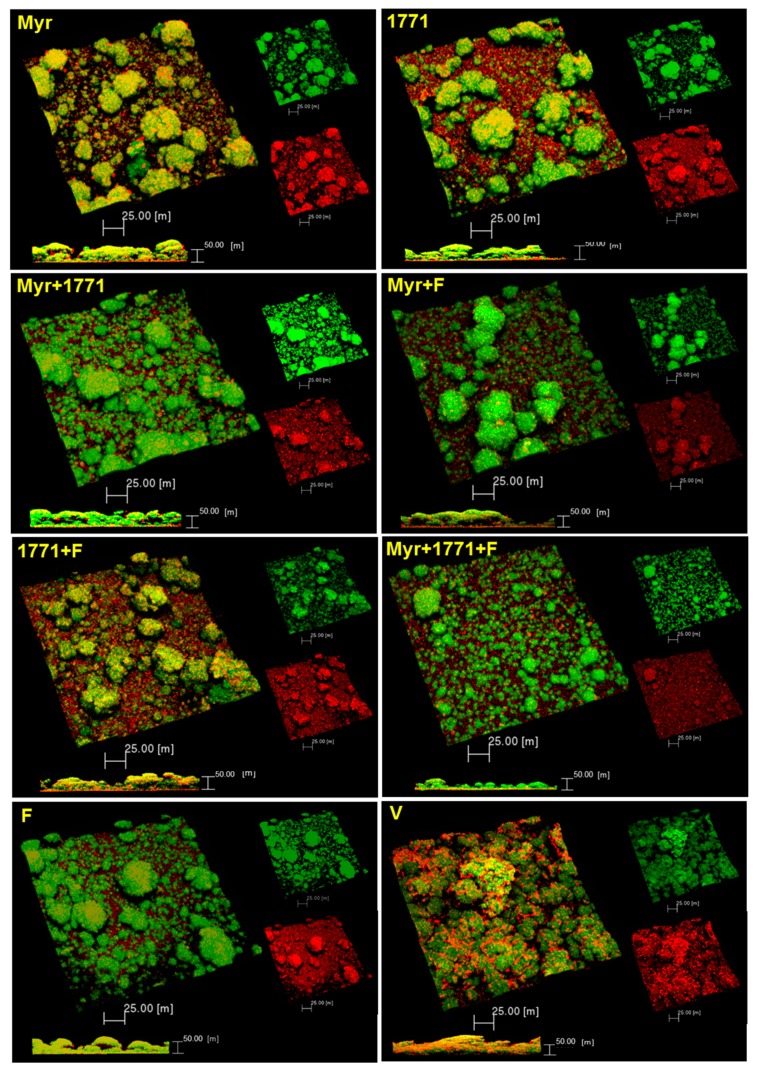
3D architecture of the topically treated *S. mutans* biofilm. Representative images of 67 h-old biofilms are displayed in this image. The red color represents exopolysaccharides produced by *S. mutans* (Alexa Fluor 647) and the green color *S. mutans* cells (SYTO 9). The larger image in each set represents the overlapping images of the red and green channels, which are shown separately in a smaller size (scale bars of 25 μm). The bottom images are a cross-section of the biofilm with overlapping images of both channels (scale bars of 50 μm).

**Figure 8:**
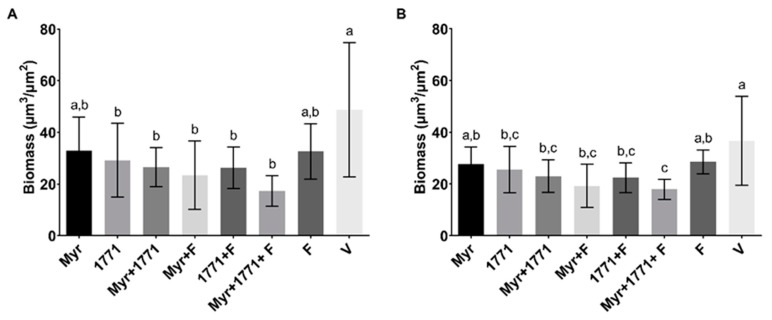
Biovolume of bacteria and EPS in topically treated biofilms. Biovolume is represented as biomass (μm^3^/μm^2^) of bacteria (**A**) and EPS (**B**). Bars with the same letters indicate no statistical difference between the different treatments in each graph (*p* > 0.05; one-way ANOVA, followed by Tukey’s test). The data represented are the means, and the error bars correspond to the standard deviation.

**Figure 9:**
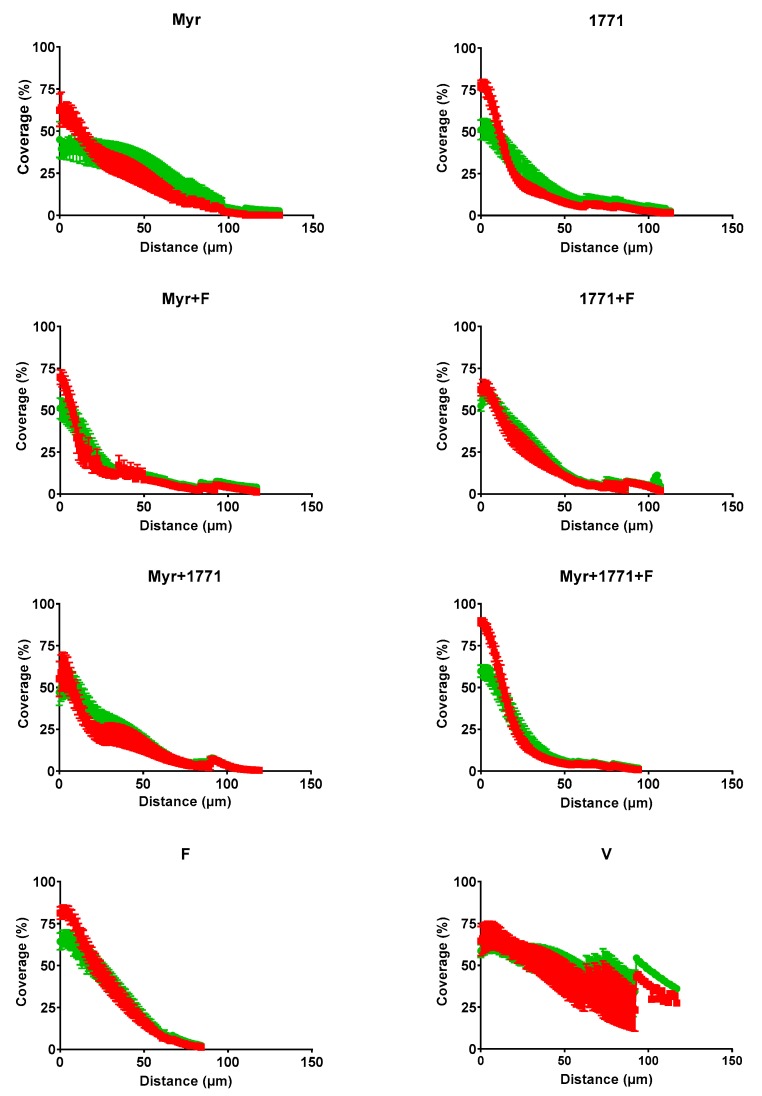
Profile of the distribution of bacteria (in green) and EPS (in red) in each of the topically treated biofilms. The data shown are the mean and standard error percentage coverage per area from the interface substratum/biofilm (hydroxyapatite disc) to the top (outer layer) of each biofilm at 67 h (n = 12 images per biofilm).

Representative images of the 3D structure of biofilms are shown in Figure 7. Biofilms treated with the vehicle presented agglomerations of well-defined large microcolonies protected by the EPS in the extracellular matrix. In contrast, the biofilms treated with the combination Myr + 1771 + F presented smaller microcolonies that were spread out across the disc surface, with a less defined EPS matrix. The treatment Myr + 1771 also yielded fewer microcolonies, but they appear to be closer together, and eventually, larger microcolonies are observed. The treatments Myr, 1771, Myr + F, and 1771 + F presented a similar architecture, with large microcolonies and small microcolonies (interspaced between the larger ones) spread out on the surface of the disc. Finally, fluoride-treated biofilms (F) showed agglomerations of microcolonies similar to the vehicle-treated biofilms (V), but smaller in size. Thus, in general, the 3D structures of the treated biofilms were different, but the most striking differences were observed for biofilms treated with the combination of Myr + 1771 + F versus the vehicle-treaded. This observation can be reinforced by the amount of biovolume (biomass) of bacteria and EPS (Figure 8) and the distribution profile of bacteria and EPS (Figure 9). The percentage of coverage per area from the interface substrate/biofilm (hydroxyapatite disc) to the top (outer layer) of each biofilm demonstrated that the vehicle exhibited a higher percentage of coverage compared to the other treatments, especially the combination of Myr + 1771 + F.

#### 3.3.5. *S. mutans* Gene Expression

**Figure 10:**
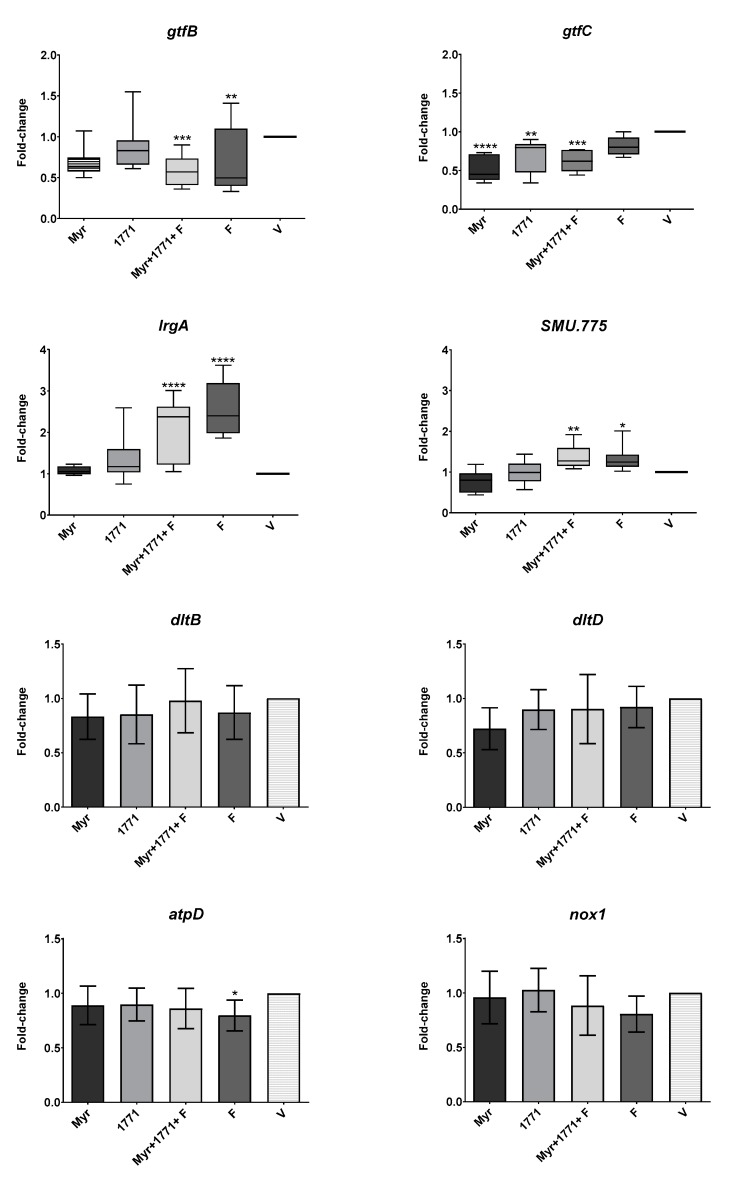
Gene expression profile of *S. mutans* biofilms after topical treatments. The fold-change is relative to vehicle-treated biofilms. Data presented are median and interquartile range are shown for genes *gtfB*, *gtfC*, *IrgA*, and *SMU.775* (box plot graphs, Kruskal–Wallis test, followed by Dunn post-test), while mean and standard deviation for genes *dltB*, *dltD*, *atpD*, and *nox1* (bar graphs; one-way ANOVA, followed by Tukey’s test). The asterisks depict significant statistical differences of treatments versus the vehicle, where * *p* ≤ 0.05, ** *p* ≤ 0.01, *** *p* ≤ 0.001, and **** *p* ≤ 0.0001. The data were obtained from three experiments (with two cDNA per experiment), and the quantification of qPCR expression was performed in duplicate.

The effect of five selected treatments on *S. mutans* gene expression is depicted in Figure 10. The *gtfB* and *gtfC* genes that encode exoenzymes that synthesizes exopolysaccharides presented lower expression in biofilms treated by the combination of Myr + 1771 + F (vs. V; *p* ≤ 0.0005; Kruskal–Wallis test, with Dunn’s post-test). However, the expression of *gtfB* was also repressed by fluoride (*p* ≤ 0.0092), while the expression of *gtfC* was down-regulated by Myr and 1771 alone. In contrast, *lrgA* and *SMU.775* genes were induced by the combination of Myr + 1771 + F and F alone (vs. V; *p* ≤ 0.0005; Kruskal–Wallis test, with Dunn’s post-test). Nonetheless, the expression of *lrgA* and *SMU.775* were similar for biofilms treated with compound 1771 alone, myricetin alone, and vehicle-control (*p* > 0.05). In addition, there were no significant differences in the expression of genes *nox1*, *dltB*, and *dltD* genes for the tested treatments and vehicle. Nevertheless, the fluoride alone significantly decreased the expression of *atpD*, involved in *S. mutans* acid tolerance.

### 3.4. Cytotoxicity of Treatments

**Figure 11:**
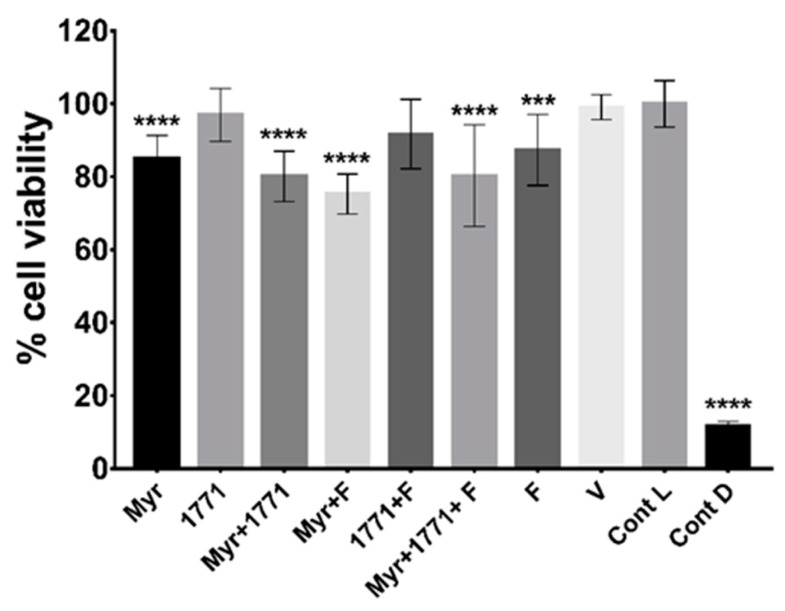
Cell viability of keratinocytes NOK-si after exposure to treatments. The data represented are the means, and the error bars correspond to the standard deviation “Cont L” indicates cell viability control and “Cont D”, the cell death control. The percentage of cell viability was obtained considering the cell viability control as 100%. The asterisks denote a statistically significant difference of a specific extract versus cell viability control (Cont L), where **** *p* < 0.0001 and *** *p* = 0.0005 (one-way ANOVA, followed by Tukey’s test).

The exposure of NOK-si lineage cells for 1 h to the four treatments containing myricetin and fluoride alone decreased cell viability by 13% to 25%, as compared to the viability control (Figure 11). Nevertheless, the model used here had cells organized as a monolayer and not a tissue with a three-dimensional organization seen in the oral mucosa. Furthermore, a possible formulation to be used in the oral cavity will have a shorter exposure time, as tested here for topical treatments of biofilms (1.5 min).

## 4. Discussion

Approaches combining bioactive agents with fluoride for preventing cariogenic biofilm development have been proposed, including compounds that affect the production of an EPS-rich matrix [22,37]. Because EPS interacts with eDNA and LTA the extracellular matrix [9] and meddling with genes associated with EPS, eDNA and LTA reduces the cariogenicity of *S. mutans* [13], the current study investigated whether a compound that targets LTA metabolism in Gram-positive bacteria and works as an antimicrobial [23,24] would be effective for topical treatment and whether combining this agent with a flavonoid that hinders EPS production and fluoride would have a more pronounced effect in hampering *S. mutans* biofilm development.

The topical application of compound 1771 and myricetin alone prevented the accumulation of *S. mutans* biofilm in vitro. However, the effect was more accentuated in the polystyrene plate model in which the agents were present for 24 h and not 1.5 min. The effect of compound 1771 could be because it had an antimicrobial effect [23], inhibiting bacterial growth in the biofilm, and by causing an alteration in the bacterial cell surface (change in LTA) that could hinder EPS binding. Nonetheless, the effect was more pronounced when compound 1771 was associated with myricetin and fluoride, affecting the amount of matrix components and the 3D architecture of biofilms. The combination of compound 1771 + myricetin + fluoride reduced three logs of *S. mutans* counts, ≈60% of the biomass, ≥74% water-soluble and -insoluble EPS, and ≈20% of LTA in the matrix (vs. vehicle; Figure 5 and Figure 6). Nevertheless, the slight changes in the quantities of eDNA and LTA could affect the 3D organization of the biofilms, as both biomolecules interact with EPS in the matrix at distinct stages of biofilm development; eDNA at the early phases while LTA during the later phases of biofilm development [9]. Therefore, these effects may have been caused by the interaction of the flavonoid with exoenzymes GtfB and GtfC and by repression of the genes that encoded them, decreasing EPS biosynthesis, as observed in previous studies [20,22], and 3D structure (Figure 7 and Figure 9). Moreover, the agents combined with fluoride lead to higher pH values in the spent media (vs. vehicle or agents alone or combined). At 67 h (after incubation in sucrose and starch), all treatments yielded pH values higher than the vehicle, especially the combination strategy Myr + 1771 + F (Figure 4), indicating that the bacterium acid production ability was compromised, either because these agents interfered with glycolysis or lower quantity of viable cells in these biofilms (Figure 5). Inhibition of the dynamic processes that occur within a biofilm can minimize or avoid the damage caused by it on teeth surfaces and prevent dental caries.

EPS synthesis and its 3D organization in the matrix is a determinant factor in the etiology of dental caries [7]. Here, the expression of *gtfB* and *gtfC* genes was lower in biofilms treated by the combination of Myr + 1771 + F (Figure 10). Although the magnitude of the differences was lower than 2-fold (Figure 10), biologically, the accumulative products resulting from the differences in the expression of *gtfB* and *gtfC* is shown as lower quantities of EPS, distribution of EPS, and size of microcolonies. The expression of *gtfB* was also repressed by fluoride, and the expression of *gtfC* was down-regulated by Myr and 1771 alone. These differences may be because GtfB produces water-insoluble EPS, while GtfC produces both water-soluble and -insoluble EPS, and the location where these exoenzymes bind preferentially. GtfB has more affinity to the cell wall and GtfC to the pellicle; the differences in the location of these active exoenzymes can explain the distinct pattern of 3D structure found for treated biofilms.

Moreover, repression of *gtfB* and *gtfC* may explain the reduction of water-soluble and -insoluble EPS in the matrix. As GtfB binds to the surface of the microorganisms and the surface of hydroxyapatite [38], the EPS formed on the microbial surface improves the interactions between the microorganisms, cellular aggregation, and increases the cohesion of the biofilm [39]. Of note, the expression of the genes associated with the synthesis, binding, and remodeling of exopolysaccharides is reduced in a deletion strain of the *gtfB* gene [12]. Therefore, the adverse effect on *gtfB* gene expression justifies the low content of water-insoluble EPS and biomass in biofilms treated with myricetin, compound 1771 combined or not with fluoride; resulting in a biofilm with disadvantages in the survival and persistence of virulent organisms. Fluoride makes an active contribution to the repression of *gtfB*, regardless of concentration (125 or 250 ppm F) [21].

Although eDNA quantification showed statistically significant differences for the combination of myricetin, 1771 compound with or without fluoride, compared to the effect of treatments on EPS, myricetin and compound 1771 affected the amount of eDNA to a lesser extent (Figure 6). This matrix component is important in the construction, 3D architecture, and stability of the extracellular matrix [7,9], and the virulence of *S. mutans* biofilms [13]. Surprisingly, the expression of *lrgA* was higher in biofilms treated with the combination of Myr + 1771 + F and F alone; thus, this behavior may be associated with fluoride. *lrgA* gene involved in *S. mutans* cell-wall remodeling and autolysis process that can yield eDNA [40,41]. Therefore, an increase in expression of *lrgA* may indicate that these treatments trigger cell wall remodeling and/or to autolysis. Furthermore, eDNA can be actively secreted from live *S. mutans* cells via additional metabolic pathways involved in protein secretion and components for protein insertion into membranes (i.e., Ffh, YidC1, and YidC2) [8]. However, eDNA may be release but may not be incorporated in the matrix if treatments are impeding EPS synthesis and/or binding. Consequently, eDNA, as a soluble component, could be in the spent medium [42,43], or being consumed as a nutrient during the periods with low carbohydrate concentration in the medium and not as part of the structure of biofilms thereby affecting the 3D architecture of treated biofilms (vs. vehicle control).

In addition, the LTA content in the matrix of the biofilms treated was not that different. Nonetheless, LTA quantities were significantly reduced in those subjected to the four treatments containing compound 1771 (1771 alone, 1771, Myr + 1771, 1771 + F, and Myr + 1771 + F) (Figure 6). A small reduction in LTA may also be contributing to alteration in the biofilm architecture, *S. mutans* viable population, biomass, and EPS in the matrix. Interestingly, the expression of gene *SMU.775* was induced by the combination of Myr + 1771 + F and F alone, while 1771 or myricetin alone yield similar expression to the vehicle-control. Thus, the induction of SMU.775 could be because of fluoride per se, as for *lrgA*. *SMU.775* is a hypothetical gene with homology to the LTA synthase from *S. aureus* and so, could be involved in the metabolism of LTA [12]. However, there were no significant differences in the expression of genes *dltB* and *dltD* for the tested treatments and the vehicle (Figure 10). Hence, the tested agents alone or combined with or fluoride did not affect the expression of *dltABCD operon* genes involved in the addition of D-alanine residues during LTA synthesis [44]. Several studies demonstrated that interfering with the D-alanization process of LTA affects the cell surface charge and susceptibility to antimicrobials [45,46,47]. Nevertheless, it is possible that the compound 1771 interfered with the LTA metabolism and affected the cell surface charge and/or composition, thereby interfering with how EPS would bind to cells and mediate the formation of microcolonies and EPS distribution in the 3D structure of treated biofilms.

During biofilm development, the microbial cells produce components for the extracellular matrix, and these components attach to the cell wall and to the substrate (i.e., the surface where biofilm grows on) [48]. Thus, alterations in the cell wall turnover and composition (and charge), or quality and quantity of matrix’ components, can interfere with cell-cell binding, cell-matrix, cell-matrix-dental surface, modifying the 3D architecture of biofilms and its cariogenic potential. Furthermore, the microbial cells in biofilms with distinct 3D structures may respond differently to environmental stresses. *S. mutans* possess a vast arsenal to cope with these stressors, especially acidic environment, and oxidative stresses.

Here, the expression of nox1 related to the oxidative stress response was similar between the tested treatments and vehicle control (Figure 10). However, the F slightly decreased the expression of *atpD*, involved in *S. mutans* acid tolerance (Figure 10). Fluoride affects the glycolytic activity in cells and inhibits intracellular enzymes when it interferes with membrane proton permeability [49], which would affect the tolerance of *S. mutans* to the acidic microenvironments within a biofilm [19,21]. Myricetin also contributes aciduricity via inhibition of glycolytic activity by altering membrane permeability [21]. Nevertheless, herein, when myricetin and 1771 were used alone or in combination with fluoride to treat biofilms, these agents did not interfere with the expression of *atpD*, which could be because of the concentrations used, exposure time and/or their effect would be detected at a different biofilm developmental phase. Nonetheless, it was demonstrated that insoluble glucans are an essential factor linking acidogenicity with aciduricity [50]; thereby, the combination tested here would not only influence the matrix composition and viable counts but how the cells in the biofilm respond to an acidic environment during biofilm growth.

The combination strategy yielded lower EPS matrix production and the number of small microcolonies (Figure 7, Figure 8 and Figure 9). The morphology of the microcolonies and EPS distribution can influence the diffusion of the metabolites and the action of agents in biofilms. Within larger microcolonies, acidic niches are created, with pH values 4.5–5.5, and cell death by cationic chlorhexidine is decreased [6]. The persistence of these acidic microenvironment leads to dental demineralization. Thus, the use of myricetin and compound 1771 combined with fluoride could be an ally in reducing the pathogenicity of the dental biofilm. Usage of myricetin and compound 1771 is not intended to eliminate fluoride but to improve the cariostatic efficacy of this product due to the advantages. Although the *S. mutans* biofilm model employed here for twice-daily topical treatments does not mimic the biological interaction of microorganisms within a cariogenic dental plaque, it shows that the tested agents interfered with critical cariogenic properties. Future studies should dissect how the proposed combination of Myr + 1771 + F affects the complex oral microbiota and prevents carious lesions in vivo, and whether increasing the concentration of agents and/or exposure/retention time (e.g., via drug-delivery systems) could improve the effectiveness of this combination.

In summary, the topical application of a compound that modulates lipoteichoic acid metabolism prevented the accumulation of *S. mutans* biofilm in vitro. Moreover, the effect was more pronounced when compound 1771 was associated with fluoride and a flavonoid. Nevertheless, the effects of a formulation with the three agents combined should be further tested in preclinical studies, testing distinct concentration to potentiate the effect to uncover its therapeutic potential fully.

## Supplementary Materials

The Supplementary Materials are available online.
