# Supplementary material for: Modulation of Lipoteichoic Acids and Exopolysaccharides Prevents Streptococcus mutans Biofilm Accumulation
Source: Molecules. 2020 May 9;25(9):2232. doi: 10.3390/molecules25092232 (PMC7249192; doi:10.3390/molecules25092232)
Supplement: Supplementary file 1 [file molecules-25-02232-s001.pdf]

Modulation of lipoteichoic acids and exopolysaccharides prevents *Streptococcus mutans* biofilm accumulation

Supplementary Information

Tables with tabulated results are presented for Figures depicting graphs in the main document.

Table S1. Antimicrobial Activity of Myricetin (A) and Compound 1771 (B): data shown in Figure 2 as CFU/mL(log).

(A) Myricetin

	500 µg/mL	250 µg/mL	125 µg/mL	62.5 µg/mL	0 µg/mL (V)	*S. mutans*
Mean	2,908	4,861	8,489	8,373	8,710	8,512
Std. Deviation	0,4161	0,4965	0,09868	0,2486	0,1076	0,06922

(B) Compound 1771

	166.7 µg/mL	83.35 µg/mL	41.675 µg/mL	20.84 µg/mL	10.42 µg/mL	5.21 µg/mL	2.605 µg/mL	1.302 µg/mL	0.651 µg/mL	0.3255 µg/mL	0 µg/mL (V)	*S. mutans*
Mean	3,946	3,822	4,262	4,556	4,503	4,652	4,718	4,635	5,549	8,537	8,519	8,521
Std. Deviation	0,4843	0,5453	0,4776	0,09482	0,2923	0,05589	0,1475	0,07116	0,5293	0,2134	0,2937	0,2347

Table S2. Antibiofilm Activity Using the Polystyrene Microplate Model: data shown in Figure 3 as *S. mutans* viable population—CFU/mL(log) (A) and biomass—%OD_{570nm} (B).

		Myr 2xMIC	Myr MIC	V (Myr)	1771 2xMIC	1771 MIC	V (1771)	*S. mutans*
(A) S. mutans viable population—CFU/mL(log)	Minimum	0,000	0,000	6,000	2,300	2,000	6,000	6,000
	25% Percentile	0,000	0,000	6,435	2,780	2,480	6,300	6,300
	Median	0,000	0,000	6,700	2,900	2,780	6,700	7,280
	75% Percentile	1,110	2,225	6,900	3,133	3,018	6,850	7,395
	Maximum	2,150	2,780	7,000	3,380	3,230	7,000	7,430
(B) Biomass—%OD_{570nm}	Minimum	0,06000	0,000	57,73	0,05000	0,05000	56,54	48,25
	25% Percentile	0,3000	0,1350	71,77	0,1100	0,1750	71,48	81,32
	Median	0,3900	0,3000	114,6	0,3550	0,4650	106,6	103,3
	75% Percentile	0,5850	0,4800	124,1	0,9175	2,140	120,2	122,7
	Maximum	0,8900	0,6000	136,4	2,360	5,390	126,9	145,1

Myr: myricetin. 1771: compound 1771. V: vehicle used for each compound. MIC: minimum inhibitory concentration.

Table S3. pH of spent culture media from topically treated biofilms at distinct developmental phases: data shown in Figure 4.

**Data per time point**		**Myr**	**1771**	**Myr+1771**	**Myr+F**	**1771+F**	**Myr+1771+ F**	**F**	**V**
**19 h**	Mean	5,8042	5,9022	5,7930	5,6747	5,6827	5,6912	5,7600	5,7365
	Std. Deviation	0,1242	0,0674	0,1142	0,2440	0,1286	0,2162	0,1611	0,1478
**27 h**	Mean	5,4593	5,4500	5,4947	5,4098	5,4427	5,1782	5,3920	5,3665
	Std. Deviation	0,0503	0,1267	0,0903	0,1106	0,0380	1,4427	0,1103	0,0975
**43 h**	Mean	4,6765	4,6765	4,6543	4,7797	4,7007	4,8585	4,6113	4,3413
	Std. Deviation	0,0524	0,0205	0,0311	0,0946	0,0467	1,0285	0,0490	0,0441
**51 h**	Mean	5,4390	5,4067	5,4208	5,3720	5,4950	4,8424	5,3973	5,1930
	Std. Deviation	0,0860	0,0874	0,0962	0,1596	0,0331	1,1536	0,0850	0,0396
**67 h**	Mean	5,0578	4,9503	5,0277	5,1602	5,3238	5,1464	5,3658	4,4845
	Std. Deviation	0,1270	0,0362	0,1338	0,1796	0,0416	1,1749	0,0934	0,0779

Table S4. *S. mutans* viable counts (A) and insoluble biomass (dry-weight) (B) of topically treated biofilms: data shown in Figure 5.

		Myr	1771	Myr+1771	Myr+F	1771+F	Myr+1771+ F	F	V
(A) *S. mutans* viable counts—CFU/mL(log)	Mean	6,840	8,095	7,779	7,025	6,965	5,790	6,952	8,870
	Std. Deviation	0,3099	0,2278	0,2146	0,1841	0,2293	0,2283	0,2832	0,2575
(B) Insoluble biomass—mg	Mean	2,737	3,273	3,156	2,873	2,848	1,654	2,856	4,090
	Std. Deviation	0,7325	0,4864	0,1917	0,4383	0,2401	0,1982	0,1645	0,3950

Table S5. The extracellular matrix components in the topically treated biofilms: data shown in Figure 6.

Matrix component		Myr	1771	Myr+1771	Myr+F	1771+F	Myr+1771+ F	F	V
(A) Water-soluble EPS—µg	Mean	77,76	116,7	83,63	96,82	65,16	58,37	118,6	223,5
	Std. Deviation	10,23	38,09	16,93	12,59	17,65	24,20	18,66	35,28
(B) Water-insoluble EPS—µg	Mean	232,0	505,2	539,5	334,7	414,8	141,5	500,1	1134
	Std. Deviation	59,29	137,1	113,3	106,4	38,80	49,39	43,65	72,73
(C) eDNA—ηg	Mean	187,5	219,1	174,3	186,7	181,7	186,6	211,8	220,4
	Std. Deviation	33,80	31,27	28,63	18,59	19,42	31,94	38,27	58,74
(D) LTA—µg	Mean	77,76	116,7	83,63	96,82	65,16	58,37	118,6	223,5
	Std. Deviation	10,23	38,09	16,93	12,59	17,65	24,20	18,66	35,28

Table S6. Biovolume of bacteria and EPS in topically treated biofilms: data shown in Figure 8. Biovolume is represented as biomass ($\mu m^3/\mu m^2$) of bacteria (A) and EPS (B).

		Myr	1771	Myr+1771	Myr+F	1771+F	Myr+1771+ F	F	V
Bacteria (A)	Mean	33,01	29,23	26,50	23,38	26,31	17,33	32,67	48,72
	Std. Deviation	12,99	14,26	7,544	13,25	8,009	5,874	10,73	26,04
EPS (B)	Mean	27,64	25,62	23,05	19,28	22,45	17,93	28,60	36,75
	Std. Deviation	6,661	8,974	6,323	8,328	5,747	3,839	4,609	17,22

Table S7. Data used to construct the profile of the distribution of bacteria and EPS in each of the topically treated biofilms. The data shown are the mean (and error) percentage coverage per area from the interface substratum/biofilm (hydroxyapatite disc— 0 μm) to the top (outer layer) of each biofilm at 67 h: data shown in Figure 9.

Treatment	Myr				1771				Myr+1771				Myr+F				1771+F				Myr+1771+F				F				V			
Distance (μm)	Bacteria		EPS		Bacteria		EPS		Bacteria		EPS		Bacteria		EPS		Bacteria		EPS		Bacteria		EPS		Bacteria		EPS		Bacteria		EPS	
	Mean	SEM	Mean	SEM	Mean	SEM	Mean	SEM	Mean	SEM	Mean	SEM	Mean	SEM	Mean	SEM	Mean	SEM	Mean	SEM	Mean	SEM	Mean	SEM	Mean	SEM	Mean	SEM	Mean	SEM	Mean	SEM
**0**	45,0	10,6	62,4	9,6	51,1	5,8	77,9	2,9	47,1	7,8	55,1	10,4	51,1	6,1	69,7	4,3	52,6	3,3	62,3	3,6	59,7	3,6	89,3	2,3	64,4	5,0	81,4	3,7	58,6	3,9	64,6	8,7
**1**	39,5	6,0	63,8	9,3	51,5	5,9	77,9	3,3	50,1	8,3	58,7	10,8	50,5	6,3	68,9	5,1	56,3	3,2	64,2	4,1	59,8	3,8	89,0	2,4	64,8	5,0	81,6	3,9	59,8	4,0	65,8	8,7
**2**	39,2	4,7	59,2	6,9	51,9	6,0	77,5	3,5	51,4	8,5	60,2	10,8	49,9	6,5	67,2	5,5	56,6	3,0	63,4	4,4	59,5	4,2	87,6	2,9	66,4	4,8	82,1	4,1	60,4	4,2	66,6	8,7
**3**	40,1	4,7	59,4	6,9	51,2	6,3	75,3	4,7	52,1	8,4	59,9	10,8	49,2	6,5	64,7	5,6	58,5	3,1	63,3	4,6	58,9	4,5	85,4	3,2	66,1	4,9	82,1	3,8	61,7	4,3	66,9	8,9
**4**	38,8	5,7	60,1	7,1	51,1	6,3	74,4	4,8	52,5	8,4	58,7	10,6	48,3	6,8	61,6	6,5	58,3	3,1	61,9	4,8	58,4	4,6	84,5	3,4	66,5	4,9	82,2	3,7	61,3	4,2	67,7	8,7
**5**	41,3	5,1	59,5	7,2	49,9	6,4	71,2	5,5	52,5	8,3	57,6	10,5	47,1	6,7	57,9	6,3	58,2	3,0	60,6	5,1	56,9	5,0	80,4	4,2	65,7	5,1	81,2	4,3	61,7	4,4	67,8	8,6
**6**	39,0	6,1	58,3	7,0	49,3	6,4	69,3	5,9	52,2	8,1	55,0	10,0	46,1	6,9	54,6	7,0	57,8	2,9	58,6	4,7	56,0	5,2	78,5	4,3	64,7	5,2	79,1	4,6	62,3	4,1	67,6	8,5
**7**	41,8	5,5	58,2	7,1	47,7	6,6	65,9	5,5	51,5	8,0	52,2	9,6	45,0	7,0	51,8	7,6	56,7	3,2	56,8	5,4	54,7	5,5	75,5	5,1	64,5	5,5	79,1	4,1	61,9	4,4	68,0	8,3
**8**	38,7	6,6	56,9	7,0	47,0	6,6	62,3	5,9	51,1	7,6	50,1	9,1	44,0	7,2	47,4	7,3	55,6	3,0	54,1	4,7	52,2	5,7	70,9	5,5	61,4	5,7	75,7	4,8	62,0	4,0	67,7	7,9
**9**	42,1	5,8	54,9	6,6	46,0	6,6	59,4	5,4	49,8	7,3	46,2	8,5	42,7	7,3	44,4	7,7	54,3	3,2	52,0	5,0	51,6	5,8	69,2	5,6	62,6	5,9	76,0	4,7	61,7	4,5	66,9	7,8
**10**	38,8	6,9	54,1	6,6	44,5	6,8	54,4	5,1	49,2	7,2	44,7	8,3	41,3	7,3	33,6	9,3	53,2	3,4	50,2	5,0	49,1	6,3	62,4	6,0	60,8	6,2	73,2	4,9	61,8	4,0	66,6	7,4
**11**	42,4	5,9	51,4	6,1	43,6	6,9	51,6	4,8	47,9	6,7	41,1	7,3	40,5	7,3	29,9	9,7	51,4	3,4	47,1	4,7	47,8	6,4	59,8	6,3	60,0	6,4	69,5	5,7	60,8	4,5	65,9	7,2
**12**	38,1	7,2	50,6	5,9	42,0	6,8	47,1	4,2	46,4	6,6	38,7	7,0	38,2	6,9	27,2	8,6	50,2	3,9	45,9	5,1	45,9	6,6	56,6	6,9	56,6	6,2	66,7	5,8	61,5	4,0	65,7	6,9
**13**	42,1	6,3	49,4	5,8	41,4	6,8	43,5	3,9	45,6	6,2	36,6	6,4	37,0	7,1	26,2	8,5	48,3	4,1	43,0	5,0	43,4	6,8	50,9	6,9	54,6	6,2	66,0	6,1	59,9	4,5	64,3	6,5
**14**	39,3	7,0	46,8	5,4	40,4	6,8	39,9	2,7	43,6	5,9	33,4	5,6	36,3	7,1	24,0	7,3	46,9	4,3	41,5	5,2	42,6	6,8	48,4	6,6	56,7	6,8	64,8	6,3	59,8	4,8	63,5	7,0
**15**	37,9	7,5	46,5	5,3	39,1	7,0	36,3	2,6	42,9	5,9	32,4	5,4	34,5	7,0	22,5	6,9	45,8	4,6	40,1	5,3	39,5	6,9	43,1	6,8	55,3	6,9	61,4	6,3	60,3	4,3	63,1	6,1
**16**	37,3	7,7	44,1	5,0	37,9	7,0	33,5	1,9	41,2	5,4	29,7	4,7	32,5	6,5	21,6	6,4	43,9	4,8	38,1	5,4	38,2	6,9	39,9	6,4	51,3	6,3	59,7	6,6	60,1	4,4	62,6	6,0
**17**	38,9	7,4	43,1	4,9	36,7	7,1	30,6	1,4	39,9	5,3	28,4	4,5	31,4	6,4	26,6	6,9	43,1	5,0	37,3	5,6	36,8	7,0	36,6	6,1	53,7	7,0	57,7	6,4	58,7	5,2	62,2	5,8
**18**	37,2	7,9	41,6	5,1	35,3	7,1	28,4	1,8	38,9	5,1	27,2	4,5	29,9	6,0	20,8	4,8	41,4	5,3	35,6	5,7	34,1	6,8	32,4	5,8	52,3	7,0	55,4	6,6	59,2	4,7	59,9	6,4
**19**	38,1	7,5	39,9	4,8	34,5	7,1	26,3	1,3	37,1	4,8	25,5	4,4	28,0	5,4	17,0	4,4	40,4	5,4	34,8	5,8	33,4	6,8	30,5	5,2	51,8	7,0	54,4	6,6	58,1	5,5	61,1	5,8

**20**	38,0	7,7	39,2	5,0	33,2	7,3	24,4	1,9	36,6	4,8	25,1	4,5	26,8	4,9	16,5	4,0	39,5	5,5	33,9	5,9	31,0	6,6	27,1	4,9	50,3	6,9	51,9	6,4	57,4	5,7	60,1	5,8
**21**	36,8	8,0	37,6	5,1	31,9	7,1	22,9	2,1	35,0	4,6	23,9	4,8	25,1	4,5	16,2	3,6	38,2	5,5	32,7	5,9	29,5	6,4	25,1	4,6	49,5	6,9	50,7	6,5	58,6	5,2	59,7	5,8
**22**	36,7	8,0	37,2	5,0	31,1	7,3	21,6	2,3	34,0	4,6	23,6	5,2	24,0	3,8	21,1	5,5	37,5	5,6	32,1	5,9	28,0	6,1	23,0	4,2	48,6	6,7	49,2	6,3	58,4	5,4	57,9	6,6
**23**	37,4	7,9	36,6	5,2	30,1	7,5	20,5	2,6	33,4	4,9	22,7	4,9	22,9	3,6	16,2	2,7	36,4	5,6	30,9	5,9	26,0	5,8	20,6	3,7	47,4	6,7	47,5	6,4	56,4	6,2	58,4	6,1
**24**	36,1	8,2	35,3	5,3	28,9	7,0	19,5	2,7	32,1	4,9	22,3	5,4	21,6	2,9	14,1	2,6	35,6	5,6	30,2	5,9	25,0	5,5	19,4	3,4	46,7	6,6	46,5	6,2	56,3	6,5	58,4	6,2
**25**	36,5	8,2	34,8	5,6	28,1	7,0	18,7	2,8	31,7	5,1	22,2	5,6	20,7	2,7	13,3	2,7	34,8	5,5	29,4	5,8	23,2	5,2	17,5	3,0	45,3	6,5	44,6	6,1	57,4	6,0	57,5	6,4
**26**	36,2	8,2	33,9	5,7	27,1	6,8	18,0	2,9	30,6	5,3	22,0	5,9	19,7	2,5	14,0	2,2	33,7	5,4	28,3	5,7	22,0	4,9	16,4	2,9	44,5	6,5	43,5	6,1	57,1	6,3	57,1	6,6
**27**	36,1	8,3	33,2	5,9	26,2	6,4	17,4	3,0	30,1	5,4	21,6	5,9	18,9	2,1	12,5	2,5	33,0	5,4	27,7	5,6	20,8	4,6	15,2	2,6	43,5	6,2	42,1	5,9	55,5	7,0	55,2	7,3
**28**	36,0	8,3	32,8	6,0	25,5	6,5	16,8	2,9	29,6	5,6	21,6	6,1	18,1	2,2	13,6	2,0	31,8	5,3	26,6	5,5	19,3	4,3	13,9	2,4	42,3	6,3	40,6	5,9	54,9	7,2	56,0	6,9
**29**	35,7	8,3	32,1	6,1	24,4	5,9	16,3	3,0	28,6	5,8	22,5	6,3	17,3	2,0	12,0	2,4	31,0	5,2	25,9	5,4	18,5	4,0	13,2	2,2	41,5	6,0	39,7	5,7	54,7	7,5	54,0	7,8
**30**	35,7	8,4	31,9	6,2	23,8	5,8	15,8	2,9	28,4	5,9	22,4	6,3	16,8	2,0	13,1	1,9	30,1	5,0	25,1	5,2	17,2	3,7	12,1	2,0	40,2	5,9	37,9	5,6	56,1	7,0	55,2	7,4
**31**	35,3	8,4	31,1	6,3	23,0	5,6	15,4	2,8	27,5	6,0	22,5	6,4	16,2	2,1	12,5	2,1	29,0	4,9	24,1	5,1	16,5	3,5	11,5	1,9	39,3	5,9	37,2	5,7	54,0	7,8	54,7	7,6
**32**	35,2	8,4	30,9	6,3	22,2	5,3	15,0	2,8	27,0	6,1	22,1	6,5	15,7	2,0	11,3	2,4	28,3	4,8	23,5	4,9	15,6	3,3	10,8	1,9	38,1	5,6	36,0	5,5	53,6	8,1	54,3	7,7
**33**	35,0	8,5	30,6	6,4	21,7	5,3	14,5	2,7	26,6	6,2	22,0	6,5	15,2	2,1	11,8	2,2	27,1	4,6	22,4	4,7	14,6	3,0	10,0	1,7	37,0	5,6	34,4	5,3	55,1	7,6	53,5	8,0
**34**	34,7	8,5	30,1	6,4	20,8	4,8	14,1	2,6	25,9	6,2	21,7	6,6	14,8	2,1	12,6	1,7	26,3	4,5	21,7	4,5	14,0	2,9	9,6	1,7	36,1	5,3	33,8	5,3	54,9	7,8	53,3	8,2
**35**	34,6	8,5	29,9	6,5	20,2	4,7	13,6	2,5	25,5	6,2	21,5	6,5	14,5	2,1	17,2	5,7	25,4	4,2	20,9	4,2	13,1	2,7	9,0	1,6	34,7	5,2	31,9	5,0	52,5	8,6	52,5	8,4
**36**	34,1	8,6	29,3	6,5	19,5	4,5	13,2	2,4	24,8	6,3	21,1	6,5	14,2	2,1	12,8	1,6	24,4	4,1	20,0	4,1	12,5	2,6	8,5	1,6	33,8	5,1	31,2	5,0	52,0	8,8	52,0	8,6
**37**	34,0	8,6	29,0	6,5	18,9	4,3	12,8	2,3	24,4	6,3	21,1	6,5	13,8	2,1	11,9	2,0	23,7	3,9	19,4	3,8	11,9	2,4	8,1	1,6	32,6	4,9	30,0	4,9	53,8	8,4	51,5	8,8
**38**	33,6	8,6	28,6	6,5	18,3	4,2	12,3	2,1	23,8	6,2	20,7	6,4	13,6	2,1	15,4	4,6	22,5	3,7	18,5	3,5	11,2	2,3	7,6	1,5	31,5	4,9	28,6	4,7	53,1	8,6	50,6	9,1
**39**	33,3	8,6	28,0	6,5	17,6	3,8	12,0	2,0	23,2	6,2	20,3	6,4	13,3	2,1	10,9	2,0	21,8	3,6	17,8	3,4	10,8	2,2	7,3	1,5	30,9	4,9	28,1	4,7	51,1	9,2	50,3	9,2
**40**	33,1	8,6	27,7	6,5	17,1	3,7	11,5	1,8	22,9	6,1	20,0	6,3	13,1	2,1	10,4	2,0	21,0	3,2	17,1	3,1	10,1	2,1	6,9	1,5	29,2	4,7	26,2	4,4	52,3	9,0	49,4	9,4
**41**	32,6	8,6	27,0	6,5	16,5	3,6	11,1	1,7	22,2	6,0	19,7	6,2	12,9	2,1	12,0	1,7	20,0	3,1	16,2	3,0	9,7	2,0	6,6	1,4	28,5	4,7	25,5	4,4	50,0	9,6	48,7	9,6
**42**	32,4	8,6	26,7	6,4	16,0	3,4	10,8	1,7	21,8	6,0	19,4	6,2	12,6	2,1	14,4	4,4	19,3	2,9	15,7	2,8	9,3	1,9	6,3	1,4	27,3	4,5	24,4	4,3	51,5	9,2	48,2	9,7
**43**	32,0	8,6	26,1	6,4	15,5	3,3	10,3	1,5	21,1	5,7	18,8	5,9	12,5	2,2	11,9	1,9	18,4	2,7	14,9	2,5	8,7	1,9	5,9	1,4	25,9	4,3	23,0	4,1	48,9	10,0	47,3	10,0
**44**	31,6	8,6	25,6	6,4	14,9	3,1	10,0	1,4	20,5	5,7	18,4	5,9	12,1	2,1	11,5	2,3	17,7	2,6	14,3	2,5	8,4	1,8	5,8	1,4	25,4	4,3	22,5	4,0	50,6	9,5	46,9	10,0
**45**	31,3	8,6	25,1	6,3	14,4	3,0	9,5	1,3	20,2	5,5	18,0	5,7	12,0	2,2	9,0	2,0	16,9	2,3	13,7	2,1	7,9	1,7	5,4	1,4	23,6	4,0	20,8	3,7	49,9	9,8	46,0	10,3

**46**	30,7	8,6	24,4	6,3	13,9	2,9	9,2	1,2	19,5	5,3	17,4	5,5	11,7	2,2	13,8	3,7	16,0	2,3	13,0	2,1	7,6	1,7	5,2	1,3	22,9	3,9	20,2	3,7	49,2	9,9	45,2	10,4
**47**	30,5	8,5	24,0	6,1	13,4	2,8	8,9	1,2	19,0	5,3	17,1	5,5	11,5	2,2	11,6	2,3	15,4	2,1	12,5	2,0	7,3	1,6	5,0	1,3	21,8	3,8	19,1	3,5	48,7	10,1	44,7	10,5
**48**	30,0	8,5	23,4	6,1	12,8	2,7	8,4	1,0	18,4	4,9	16,4	5,1	11,3	2,2	12,1	3,1	14,5	1,9	11,8	1,8	6,9	1,6	4,7	1,3	20,5	3,6	17,9	3,3	47,9	10,3	43,7	10,6
**49**	29,5	8,5	22,8	6,0	12,4	2,6	8,1	1,1	17,7	4,8	15,9	5,1	11,0	2,2	12,0	3,2	13,9	1,9	11,3	1,9	6,6	1,5	4,6	1,3	20,0	3,5	17,4	3,2	47,4	10,4	43,3	10,6
**50**	29,1	8,3	22,2	5,8	11,9	2,5	7,7	1,1	17,3	4,6	15,5	4,9	10,8	2,3	8,5	1,8	13,2	1,6	10,7	1,6	6,3	1,5	4,4	1,2	18,4	3,2	15,9	2,9	46,7	10,6	42,5	10,8
**51**	28,6	8,4	21,5	5,8	11,4	2,5	7,5	1,1	16,6	4,4	14,7	4,6	10,6	2,3	8,7	1,8	12,5	1,7	10,2	1,7	6,0	1,4	4,2	1,2	17,8	3,1	15,3	2,9	46,0	10,7	41,6	10,8
**52**	28,2	8,3	21,1	5,6	11,0	2,4	7,2	1,1	16,2	4,3	14,4	4,6	10,4	2,3	8,5	1,9	11,9	1,5	9,7	1,6	5,8	1,4	4,1	1,2	16,8	2,9	14,4	2,7	45,4	10,8	40,0	11,1
**53**	27,6	8,2	20,4	5,5	10,6	2,4	6,9	1,2	15,4	3,9	13,6	4,2	10,2	2,4	8,3	1,9	11,2	1,4	9,1	1,5	5,4	1,3	3,9	1,2	15,6	2,7	13,3	2,5	44,6	11,0	40,1	11,0
**54**	27,1	8,2	19,8	5,4	10,2	2,4	6,6	1,3	14,8	3,8	13,1	4,2	9,9	2,4	8,1	1,9	10,7	1,5	8,6	1,6	5,3	1,3	3,8	1,2	15,2	2,7	12,9	2,5	44,1	11,0	39,7	11,0
**55**	26,6	8,0	19,2	5,2	9,8	2,4	6,4	1,3	14,3	3,6	12,7	4,0	9,7	2,3	7,9	1,9	10,1	1,3	8,0	1,4	5,6	1,3	4,2	1,2	13,8	2,4	11,6	2,2	43,5	11,2	39,1	11,1
**56**	26,0	8,0	18,4	5,1	9,5	2,4	6,2	1,4	13,6	3,3	11,9	3,8	9,5	2,3	7,7	1,9	9,5	1,4	7,6	1,5	5,4	1,3	4,1	1,1	13,3	2,3	11,1	2,1	42,7	11,2	37,4	11,2
**57**	25,6	7,8	18,0	4,9	9,2	2,4	6,0	1,5	13,1	3,2	11,6	3,8	9,1	2,3	7,5	1,9	9,0	1,3	7,1	1,4	5,2	1,2	3,9	1,1	12,5	2,2	10,3	2,0	42,2	11,3	36,3	11,2
**58**	24,9	7,7	17,0	4,7	8,9	2,4	5,7	1,5	12,4	2,8	10,9	3,5	9,0	2,3	7,4	1,9	8,4	1,3	6,6	1,4	5,8	1,0	4,4	1,0	11,6	2,1	9,5	1,9	41,5	11,4	36,9	11,2
**59**	24,3	7,6	16,9	4,8	8,5	2,4	5,6	1,6	11,9	2,7	10,4	3,5	8,7	2,3	7,2	2,0	8,5	1,5	7,0	1,4	5,6	1,0	4,3	1,1	11,2	2,1	9,1	1,8	40,9	11,4	35,8	11,1
**60**	23,8	7,4	16,1	4,4	8,2	2,4	5,3	1,6	11,5	2,6	9,8	3,2	8,5	2,2	7,0	1,9	7,9	1,4	6,5	1,3	5,4	0,9	4,1	1,0	10,3	2,0	8,2	1,7	40,4	11,5	35,2	11,2
**61**	23,1	7,4	15,4	4,4	8,0	2,4	5,2	1,7	10,9	2,4	9,6	3,3	8,3	2,2	6,8	1,9	7,6	1,5	6,2	1,4	5,2	0,9	4,0	1,0	8,9	2,0	6,8	1,7	39,6	11,4	33,9	11,1
**62**	22,7	7,2	14,9	4,2	8,7	2,7	5,9	1,8	10,4	2,3	9,1	3,2	8,0	2,1	6,5	1,8	7,2	1,5	5,9	1,3	5,0	0,9	3,8	1,0	9,3	2,2	7,3	1,6	39,1	11,5	33,6	11,1
**63**	22,0	7,0	14,3	4,1	9,6	3,2	6,9	1,9	9,9	2,1	8,5	3,0	7,8	2,1	6,2	1,8	6,7	1,5	5,5	1,4	5,0	1,1	4,1	1,0	8,5	2,1	6,6	1,5	43,4	12,5	39,1	11,7
**64**	21,5	7,0	13,7	4,0	9,5	3,1	6,8	1,9	9,4	2,1	8,1	2,9	7,6	2,1	6,0	1,7	6,5	1,5	5,3	1,4	4,9	1,1	4,1	1,0	8,2	2,0	6,3	1,4	42,7	12,4	37,1	11,7
**65**	20,7	6,7	13,1	3,7	9,3	3,2	6,7	2,0	9,0	2,0	7,8	2,9	7,3	2,0	5,6	1,6	6,1	1,6	4,9	1,4	4,7	1,0	3,9	1,0	7,5	1,8	5,6	1,3	42,5	12,4	36,6	11,8
**66**	20,0	6,6	12,4	3,7	9,2	3,1	6,6	2,0	8,6	1,9	7,3	2,8	7,1	2,0	5,5	1,6	6,9	1,5	5,7	1,3	4,5	1,0	3,7	1,0	7,0	1,7	5,2	1,2	41,8	12,4	36,4	11,6
**67**	19,6	6,4	10,6	3,2	9,0	3,1	6,5	2,1	8,2	1,9	7,0	2,8	6,7	1,9	5,1	1,5	6,6	1,6	5,4	1,3	5,4	0,6	4,4	0,6	8,3	0,3	6,0	0,6	42,0	12,3	34,7	11,7
**68**	19,1	6,3	11,1	3,2	8,8	3,1	6,5	2,1	7,8	1,8	6,6	2,7	6,5	1,9	4,9	1,5	6,3	1,7	5,0	1,4	5,1	0,6	4,1	0,6	7,5	0,3	5,3	0,6	41,7	15,2	37,4	13,9
**69**	18,6	6,2	11,2	3,4	8,7	3,0	6,4	2,1	7,4	1,8	6,3	2,6	6,2	1,8	4,6	1,5	6,1	1,7	4,9	1,4	5,0	0,7	4,1	0,6	7,3	0,3	5,1	0,6	40,8	15,0	36,3	13,6
**70**	17,9	6,0	10,7	3,2	8,5	3,0	6,3	2,1	6,4	1,9	6,0	2,6	5,9	1,7	4,3	1,4	5,7	1,9	4,5	1,5	4,7	0,6	3,8	0,6	6,6	0,2	4,5	0,4	40,4	15,1	35,7	13,7
**71**	17,2	5,8	9,1	2,8	8,4	3,0	6,2	2,1	6,9	1,8	5,6	2,5	5,8	1,8	4,1	1,4	5,5	1,9	4,3	1,6	4,5	0,7	3,6	0,7	6,2	0,2	4,2	0,6	39,4	15,0	35,0	13,5

**72**	16,8	5,6	9,3	2,7	8,3	3,0	6,0	2,0	5,9	1,9	5,4	2,5	5,4	1,7	3,8	1,2	5,2	2,0	4,0	1,6	4,4	0,7	3,4	0,7	5,9	0,1	3,9	0,5	38,8	14,9	33,5	13,5
**73**	16,2	5,5	9,6	3,0	8,2	3,0	5,8	2,0	6,0	1,9	5,1	2,4	5,8	1,9	4,2	1,4	4,9	2,0	3,7	1,6	4,1	0,7	3,1	0,7	5,3	0,1	3,5	0,5	40,9	19,0	35,6	17,1
**74**	15,7	5,4	7,8	2,5	8,0	2,9	5,7	2,0	5,7	1,7	4,8	2,4	5,6	1,8	4,0	1,3	5,7	2,5	4,3	2,1	4,0	0,7	3,0	0,8	5,1	0,2	3,3	0,5	39,7	18,6	34,4	16,7
**75**	15,1	5,2	7,5	2,3	7,9	2,9	5,5	1,9	5,6	1,8	4,6	2,4	5,3	1,7	3,7	1,3	7,4	2,8	5,6	2,5	3,7	0,7	2,6	0,7	4,7	0,0	2,9	0,4	39,3	18,6	34,0	16,7
**76**	14,5	5,1	7,6	2,4	7,8	2,9	5,3	1,8	5,4	1,7	4,3	2,2	5,2	1,7	3,6	1,3	7,2	2,6	5,4	2,5	3,5	0,8	2,5	0,8	4,3	0,1	2,7	0,5	38,5	18,4	33,1	16,4
**77**	16,6	5,2	8,0	2,3	7,6	2,8	5,1	1,7	5,0	1,8	4,1	2,2	4,9	1,6	3,3	1,2	6,8	3,0	5,1	2,8	3,3	0,8	2,3	0,7	4,1	0,1	2,5	0,5	37,8	18,3	32,4	16,2
**78**	15,9	5,1	8,7	2,6	7,4	2,8	4,9	1,7	5,2	1,8	3,8	2,1	4,7	1,6	3,1	1,2	6,5	2,8	5,0	2,8	3,7	0,8	2,6	0,9	3,7	0,1	2,2	0,4	37,4	18,3	32,0	16,1
**79**	15,4	4,9	8,5	2,6	7,2	2,7	4,8	1,6	4,9	1,8	3,5	2,1	4,5	1,6	2,9	1,2	6,2	2,9	4,8	2,9	4,4	0,0	3,4	0,0	3,5	0,1	2,1	0,5	36,3	17,8	30,9	15,6
**80**	14,7	4,7	7,7	2,3	9,0	2,4	5,9	1,0	4,5	1,9	3,4	2,0	4,2	1,5	2,6	1,1	5,9	3,1	4,6	3,0	4,1	0,0	3,0	0,0	3,2	0,1	2,1	0,2	35,9	17,8	30,5	15,6
**81**	14,1	4,6	6,4	2,1	8,8	2,3	5,7	1,0	4,5	1,8	3,1	1,9	4,1	1,5	2,5	1,2	5,7	3,0	4,5	3,0	4,0	0,0	2,9	0,0	3,1	0,1	2,0	0,2	35,0	17,4	29,7	15,3
**82**	13,7	4,4	6,2	1,8	8,5	2,2	5,5	0,8	4,8	2,2	3,2	2,3	3,8	1,4	2,2	1,1	5,4	3,2	4,3	3,2	3,8	0,0	2,6	0,0	2,9	0,1	1,8	0,2	34,3	17,2	29,0	15,0
**83**	13,5	4,5	7,6	2,6	8,4	2,2	5,3	0,8	4,4	2,1	3,0	2,1	4,2	1,7	2,6	1,3	5,2	3,2	4,2	3,2	3,6	0,0	2,4	0,0	2,5	0,1	1,6	0,2	34,1	17,2	28,7	15,0
**84**	13,3	4,5	7,3	2,5	8,1	2,0	5,2	0,7	4,2	2,1	2,8	2,1	6,9	0,7	4,5	1,5	5,0	3,2	4,1	3,2	3,5	0,0	2,3	0,0	2,3	0,0	1,3	0,0	33,0	16,7	27,7	14,5
**85**	12,5	4,0	5,4	1,5	7,9	2,0	5,0	0,6	4,8	2,4	3,3	2,5	6,5	1,0	4,2	1,7	4,8	3,3	4,0	3,3	3,2	0,0	2,0	0,0					32,7	16,6	27,4	14,4
**86**	12,2	4,1	6,1	2,0	7,6	2,0	4,9	0,5	4,5	2,2	3,0	2,3	6,4	1,1	4,1	1,8	4,6	3,2	3,8	3,2	3,1	0,0	1,9	0,0					32,1	16,3	26,6	14,1
**87**	11,8	3,8	6,4	2,3	7,4	1,8	4,7	0,4	4,3	2,2	2,9	2,2	5,9	1,6	3,8	2,1	7,7	0,0	7,1	0,0	2,9	0,0	1,7	0,0					31,5	16,1	26,0	13,8
**88**	11,2	3,5	4,4	1,4	7,3	1,9	4,5	0,4	3,8	2,1	2,6	2,0	5,8	1,6	3,7	2,2	7,4	0,0	6,9	0,0	2,7	0,0	1,5	0,0					31,4	16,0	25,7	13,8
**89**	11,0	3,5	4,4	1,4	7,0	1,7	4,3	0,3	4,7	2,5	3,1	2,6	5,6	1,9	3,6	2,3	7,3	0,0	6,8	0,0	2,6	0,0	1,4	0,0					30,7	15,3	24,8	13,3
**90**	10,2	3,2	4,7	1,6	6,9	1,7	4,1	0,2	6,3	2,5	4,4	3,6	5,3	2,2	3,4	2,4	7,2	0,0	6,7	0,0	2,3	0,0	1,2	0,0					30,7	15,1	24,5	13,2
**91**	9,9	3,2	3,9	1,3	6,7	1,6	4,0	0,2	7,9	0,0	7,2	0,0	5,2	2,2	3,3	2,3	6,9	0,0	6,4	0,0	2,2	0,0	1,1	0,0					32,9	13,5	23,9	12,9
**92**	9,4	3,1	5,2	2,1	6,4	1,5	3,7	0,1	7,7	0,0	7,1	0,0	4,9	2,5	3,1	2,4	6,9	0,0	6,4	0,0	2,0	0,0	1,0	0,0					34,4	12,8	23,3	12,6
**93**	8,9	2,9	4,8	2,1	6,3	1,5	3,6	0,1	6,9	0,0	6,3	0,0	7,4	0,0	5,4	0,0	6,6	0,0	6,0	0,0	1,9	0,0	0,9	0,0					54,4	0,0	43,0	0,0
**94**	8,7	2,9	4,5	1,8	6,0	1,4	3,4	0,1	6,5	0,0	6,0	0,0	7,4	0,0	5,1	0,0	6,5	0,0	5,9	0,0	1,8	0,0	0,8	0,0					52,4	0,0	44,6	0,0
**95**	8,1	2,7	4,3	1,9	5,9	1,4	3,2	0,2	6,2	0,0	5,7	0,0	7,4	0,0	5,1	0,0	6,3	0,0	5,8	0,0									52,2	0,0	42,7	0,0
**96**	7,8	2,6	3,2	1,2	5,7	1,3	3,1	0,3	5,4	0,0	4,9	0,0	7,2	0,0	4,7	0,0	6,0	0,0	5,4	0,0									50,9	0,0	41,6	0,0
**97**	5,1	1,5	2,4	1,7	5,5	1,3	2,9	0,4	5,3	0,0	4,7	0,0	6,8	0,0	4,6	0,0	5,9	0,0	5,3	0,0									50,2	0,0	40,6	0,0

**98**	4,8	1,4	2,2	1,6	5,4	1,3	2,8	0,4	4,5	0,0	4,0	0,0	6,8	0,0	4,5	0,0	5,6	0,0	4,9	0,0									49,9	0,0	40,3	0,0
**99**	4,7	1,4	2,1	1,5	5,9	1,6	3,0	0,6	4,2	0,0	3,7	0,0	6,4	0,0	4,2	0,0	5,5	0,0	4,7	0,0									48,2	0,0	38,8	0,0
**100**	4,5	1,4	1,8	1,4	5,8	1,5	2,9	0,6	3,9	0,0	3,5	0,0	6,3	0,0	4,2	0,0	5,3	0,0	4,5	0,0									47,9	0,0	38,5	0,0
**101**	4,2	1,4	1,7	1,3	5,6	1,4	2,7	0,5	3,3	0,0	2,9	0,0	6,1	0,0	3,9	0,0	5,2	0,0	3,9	0,0									46,8	0,0	37,4	0,0
**102**	4,1	1,5	1,5	1,1	5,3	1,3	2,6	0,6	3,1	0,0	2,8	0,0	5,8	0,0	3,8	0,0	5,3	0,0	3,8	0,0									46,1	0,0	29,8	0,0
**103**	3,9	1,3	1,4	1,1	5,2	1,3	2,5	0,5	2,6	0,0	2,3	0,0	5,8	0,0	3,7	0,0	8,3	0,0	3,2	0,0									45,8	0,0	35,0	0,0
**104**	3,7	1,4	1,3	1,0	4,9	1,2	2,3	0,6	2,3	0,0	2,1	0,0	5,4	0,0	3,4	0,0	10,4	0,0	3,0	0,0									44,5	0,0	36,6	0,0
**105**	3,6	1,3	1,1	0,8	4,9	1,2	2,2	0,6	2,2	0,0	1,9	0,0	5,3	0,0	3,4	0,0	11,2	0,0	2,7	0,0									44,3	0,0	36,3	0,0
**106**	3,4	1,4	1,1	0,8	4,8	1,2	2,0	0,5	1,7	0,0	1,5	0,0	5,2	0,0	3,1	0,0	7,2	0,0	2,0	0,0									43,1	0,0	30,0	0,0
**107**	3,0	1,8	0,9	0,7	4,6	1,2	1,9	0,6	1,6	0,0	1,5	0,0	5,0	0,0	3,0	0,0	4,7	0,0	1,8	0,0									42,5	0,0	33,8	0,0
**108**	2,8	1,7	0,8	0,6	4,5	1,2	1,7	0,5	1,3	0,0	1,2	0,0	4,9	0,0	2,9	0,0													42,2	0,0	34,8	0,0
**109**	3,1	1,2	0,8	0,6	4,3	1,2	1,5	0,6	1,2	0,0	1,1	0,0	4,9	0,0	2,6	0,0													40,9	0,0	28,6	0,0
**110**	4,2	0,0	0,2	0,0	4,2	1,2	1,4	0,6	1,1	0,0	1,1	0,0	4,6	0,0	2,6	0,0													40,6	0,0	28,9	0,0
**111**	4,1	0,0	0,2	0,0	2,9	0,0	1,8	0,0	0,9	0,0	0,9	0,0	4,6	0,0	2,3	0,0													39,5	0,0	30,6	0,0
**112**	4,0	0,0	0,2	0,0	2,7	0,0	1,8	0,0	0,9	0,0	0,9	0,0	4,5	0,0	2,3	0,0													39,0	0,0	27,9	0,0
**113**	3,8	0,0	0,2	0,0	2,7	0,0	1,5	0,0	0,7	0,0	0,8	0,0	4,4	0,0	2,1	0,0													38,8	0,0	33,1	0,0
**114**	3,8	0,0	0,2	0,0					0,7	0,0	0,7	0,0	4,2	0,0	1,9	0,0													37,7	0,0	32,9	0,0
**115**	3,7	0,0	0,2	0,0					0,6	0,0	0,7	0,0	4,2	0,0	1,8	0,0													37,3	0,0	31,4	0,0
**116**	3,6	0,0	0,2	0,0					0,5	0,0	0,6	0,0	4,1	0,0	1,4	0,0													36,7	0,0	31,7	0,0
**117**	3,6	0,0	0,2	0,0					0,5	0,0	0,6	0,0	4,1	0,0	1,2	0,0													36,1	0,0	27,5	0,0
**118**	3,4	0,0	0,2	0,0					0,5	0,0	0,5	0,0																				
**119**	3,3	0,0	0,2	0,0					0,4	0,0	0,4	0,0																				
**120**	3,3	0,0	0,2	0,0																												
**121**	3,2	0,0	0,1	0,0																												
**122**	3,1	0,0	0,1	0,0																												
**123**	3,0	0,0	0,1	0,0																												

**124**	3,0	0,0	0,1	0,0																												
**125**	2,9	0,0	0,1	0,0																												
**126**	2,9	0,0	0,1	0,0																												
**127**	2,8	0,0	0,1	0,0																												
**128**	2,8	0,0	0,1	0,0																												
**129**	2,7	0,0	0,1	0,0																												
**130**	2,7	0,0	0,1	0,0																												

Table S8. Data used to show the gene expression profile of *S. mutans* biofilms after topical treatments. The fold-change is relative to vehicle-treated biofilms. Data presented are median and interquartile range are shown for genes *gtfB*, *gtfC*, *IrgA*, and *SMU.775*, while mean and standard deviation for genes *dltB*, *dltD*, *atpD*, and *nox1*: **data shown in Figure 10**

		Myr	1771	Myr+1771+ F	F	V
gtfB	Minimum	0,5000	0,6100	0,3600	0,3300	1,000
	25% Percentile	0,5750	0,6600	0,4100	0,4000	1,000
	Median	0,6300	0,8300	0,5700	0,4950	1,000
	75% Percentile	0,7450	0,9550	0,7325	1,098	1,000
	Maximum	1,070	1,550	0,9000	1,410	1,000
gtfC	Minimum	0,3400	0,3400	0,4400	0,6700	1,000
	25% Percentile	0,3800	0,4775	0,4925	0,7100	1,000
	Median	0,4500	0,7950	0,6200	0,8000	1,000
	75% Percentile	0,7100	0,8400	0,7650	0,9250	1,000
	Maximum	0,7300	0,9000	0,7700	1,000	1,000
lrgA	Minimum	0,9600	0,7500	1,050	1,860	1,000
	25% Percentile	0,9900	1,038	1,223	1,980	1,000
	Median	1,050	1,170	2,375	2,400	1,000
	75% Percentile	1,178	1,593	2,618	3,185	1,000
	Maximum	1,230	2,590	3,010	3,620	1,000
SMU.775	Minimum	0,4400	0,5700	1,080	1,020	1,000
	25% Percentile	0,5000	0,7775	1,155	1,133	1,000
	Median	0,8000	0,9900	1,275	1,245	1,000
	75% Percentile	0,9675	1,205	1,590	1,428	1,000
	Maximum	1,190	1,440	1,920	2,010	1,000
dltB	Mean	0,8320	0,8525	0,9783	0,8692	1,000
	Std. Deviation	0,2086	0,2701	0,2954	0,2469	0,000
dltD	Mean	0,7217	0,8975	0,9025	0,9213	1,000
	Std. Deviation	0,1922	0,1827	0,3173	0,1895	0,000
atpD	Mean	0,8900	0,8967	0,8600	0,7973	1,000
	Std. Deviation	0,1782	0,1502	0,1843	0,1416	0,000
nox1	Mean	0,9580	1,027	0,8842	0,8058	1,000
	Std. Deviation	0,2417	0,2004	0,2734	0,1656	0,000

Table S9. Cell viability of keratinocytes NOK-si after exposure to treatments. The percentage of cell viability was obtained considering the cell viability control (Cont L) as 100%: data shown in Figure 11.

	Myr	1771	Myr+1771	Myr+F	1771+F	Myr+1771+ F	F	V	Cont L	Cont D
Mean	84,94	96,93	80,09	75,26	91,69	80,34	87,39	99,09	100,0	11,75
Std. Deviation	6,392	7,278	6,905	5,458	9,494	13,91	9,745	3,389	6,385	1,171
